# In Silico Discovery of a Novel Antiviral Scaffold for SARS-CoV‑2 Targeting the Spike Glycoprotein through the Fatty Acid Binding Pocket

**DOI:** 10.1021/acsomega.4c10519

**Published:** 2025-06-04

**Authors:** Luís Queirós-Reis, Mari Kaarbo̷, Huda Al-Baldawi, Rui Alvites, Ana Colette Maurício, Andrea Brancale, Marcella Bassetto, João R. Mesquita

**Affiliations:** 1 Abel Salazar Institute of Biomedical Sciences (ICBAS), 89239 University of Porto, Porto 4050-313, Portugal; 2 Department of Microbiology, 155272 Oslo University Hospital, Oslo 0424, Norway; 3 Department of Microbiology, 6305 University of Oslo, Oslo 0316, Norway; 4 Animal Science Study Centre (CECA), University of Porto Agroenvironment, Technologies and Sciences Institute (ICETA), Porto 4051-401, Portugal; 5 Associate Laboratory for Animal and Veterinary Science (AL4AnimalS), Lisboa 1300-477, Portugal; 6 University Institute of Health Sciences (CESPU), Avenida Central de Gandra 1317, Gandra 4585-116, Portugal; 7 University of Chemistry and Technology, Prague, 166 28 Praha, Czechia; 8 School of Pharmacy and Pharmaceutical Sciences, College of Biomedical and Life Sciences, Cardiff University, Cardiff CF10 3BN, U.K.; 9 Department of Chemistry, Faculty of Science and Engineering, Swansea University, Swansea SA2 8PP, U.K.; 10 Epidemiology Research Unit (EPIunit), Institute of Public Health, University of Porto, Porto 4050-091, Portugal

## Abstract

The key viral protein for infection by SARS-CoV-2 is the spike glycoprotein (S protein), mediating entry into host cells, which therefore represents a strong focus for the development of targeted therapeutics. In this work, we explored the fatty acid binding pocket within the S protein, which stabilizes an inactive conformation and disrupts cell recognition and infection. To explore the potential of this site as a drug target, molecular dynamics simulations were performed, followed by a docking-based virtual screening of commercial druglike compounds. This in silico procedure enabled the identification of potential inhibitors of SARS-CoV-2 cell infection, likely by stabilizing an inactive spike conformation, detected in binding assays, although further experiments are required to directly confirm this action. The antiviral effect of the virtual hits was analyzed in cell-based assays, and one molecule displayed a low micromolar activity. Starting from the best antiviral compound found, structural analogues were purchased and evaluated in antiviral assays. An increase in activity was observed for multiple analogues, with the strongest antiviral compound showing submicromolar activity and low cytotoxicity. The successful identification of a new antiviral scaffold through in silico studies might pave the way for the further development of antivirals against SARS-CoV-2 and shows the reliability of the methodologies applied.

## Graphical abstract

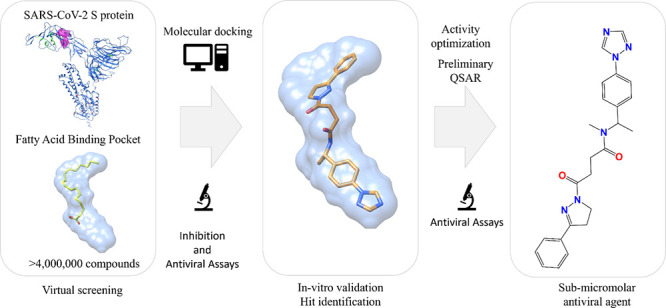

## Introduction

Coronavirus disease 19, or COVID-19, is a respiratory infection caused by severe acute respiratory syndrome coronavirus 2 (SARS-CoV-2), responsible for a global pandemic with more than 750 million people infected and seven million deaths.^1^ Coronaviruses (CoVs) are RNA viruses, belonging to the *Coronaviridae* family, that infect a wide range of domestic and wild animals.^2–5^ The name “corona” is derived from a crownlike halo observed by microscopy, formed by three major structural proteins: the spike (S), membrane, and envelope proteins, projecting from the viral envelope.^2,6,7^ CoV’s cell recognition and infection are enabled by the densely glycosylated S protein, a trimeric fusion protein that, for SARS-CoV-2, recognizes the human target angiotensin converting enzyme 2 (ACE2).^8^ Since the S protein facilitates viral entry into host cells, it is the main target for neutralizing antibodies and is a key focus in the development of therapeutics and vaccines. The S protein is composed of two subunits (S1 and S2), with S1 responsible for cell recognition and S2 containing the fusion machinery.^8,9^ A particular portion of the S1 subunit, the receptor binding domain (RBD), interacts directly with the human receptor ACE2, and it is therefore a major epitope for neutralizing antibodies.^4,9,10^ An RBD exists in each S protein monomer and can have two possible conformations: a down conformation (inactive), inaccessible for interaction with ACE2, and an up conformation (active), available for target recognition.^10,11^ Given the essential role of the S protein in infectivity, host range, and pathogenesis, any changes to its activity can have profound consequences for virus infection, with some mutations dramatically increasing the transmission and originating more dangerous viral strains.^3,10^ On the other hand, vaccines targeted at the S protein have achieved significant success,^10,12,13^ although no small-molecule inhibitors of the S protein have so far been approved for clinical use.^14^ Despite this, multiple compounds have shown the ability to affect the virus life cycle by targeting different sites in the S protein.^15–20^ One such site is the fatty acid binding pocket (FABP), which can bind fatty acids, particularly linoleic acid (LA).^18,21^ When LA binds the FABP, a stark change in S protein population dynamics is elicited, favoring the inactive conformation with all hidden RBDs.^21,22^ The S protein is formed by three independent monomers and can interact with three ACE2 molecules. Therefore, three FABPs can be identified, formed between RBDs, and composed by two clearly defined hydrophilic and hydrophobic areas (Figure 1).^21^ Critically, the FABP is conserved in human CoVs and represents a potential target for pan-coronavirus activity.^23^

**Figure 1:**
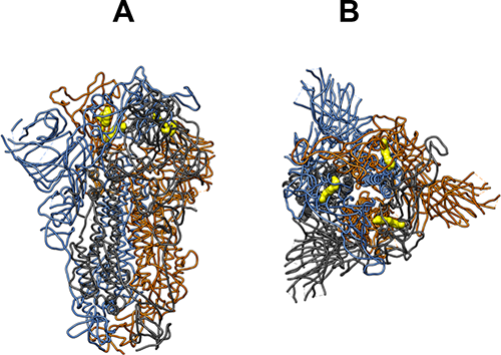
(A) Top view of the S protein in a ribbon representation with the monomers represented as blue, orange, and gray ribbons, along with the FABP in a yellow surface (PDB ID 6ZB5). (B) Side view of the S protein in a ribbon representation with the monomers represented as blue, orange, and gray ribbons, along with the FABP in a yellow surface (PDB ID 6ZB5). Abbreviations: S protein, spike glycoprotein; FABP, fatty acid binding pocket; PDB, Protein Data Bank.

These changes elicited by LA binding in the FABP significantly affect the virus life cycle, by reducing the virus–host interaction and the virus’ ability to infect new cells.^18^ Since this target site was discovered, research focused on the FABP has been significant, and multiple compounds have been identified, capable of affecting virus–host interactions.^22^ However, in addition to LA, only lifitegrast and experimental compound SPC-14 have been confirmed to bind this pocket.^16,24^ Dexamethasone, multiple fatty acids, retinoids, and liposoluble vitamins A and K have biological assays showing the ability to affect the S protein–ACE2 interaction, while despite computational studies predicting FABP interaction, explicit binding was not confirmed.^15,25,26^

Regardless of the extensive research focused on the FABP, most bioactive compounds have limitations such as fatty acids and retinoids, which display inadequate properties for translational or clinical applications. In this study, our main objective was to explore the FABP as a drug target and to identify new bioactive scaffolds more suitable for drug development. To achieve this, the binding between the FABP and fatty acids was analyzed by molecular dynamics, followed by a docking-based virtual screening of a library of commercial, druglike compounds. The virtual hits identified were then assessed in *in vitro* inhibition assays (S-ACE2 interaction) and cell-based antiviral assays. An antiviral hit molecule was found with an EC_50_ value in the low micromolar range. The subsequent evaluation of structural analogues enabled the identification of an antiviral compound with increased potency in the submicromolar range and a preliminary evaluation of structure–activity relationships for this novel antiviral scaffold.

## Results and Discussion

### S Protein Dynamics and the FABP

To explore the FABP modulatory activity on the spike protein and key binding interactions in this site, the full-length 3D structure of the S protein in the presence and absence of LA (Protein Data Bank^27^ accession code PDB IDs 7DF3, 6VYB, and 6ZB5 ^3,21,28^ ) was analyzed via molecular dynamics (MD). Three different simulations were run in triplicate for the S protein in the open conformation, closed conformation, and closed conformation with bound LA. Analysis of root-mean-square deviation (RMSD) shows geometric convergence in MD simulations for all S protein systems at 100 ns (Figure 2). The open conformation showed the highest stability, while the presence of LA stabilized the closed conformation, achieving an intermediate stabilization level.

**Figure 2:**
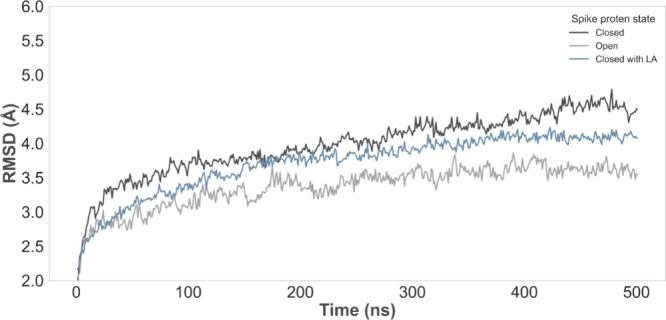
All-atom RMSD of the S protein in the closed conformation, closed conformation with LA bound, and open conformation (PDB accession codes 7DF3, 6ZB5, and 6VYB, respectively). Abbreviations: RMSD, root-mean-square deviation; S protein, spike glycoprotein; LA, linoleic acid; PDB, Protein Data Bank.

This stabilization is likely linked to the pattern of root-mean-square fluctuation (RMSF): the S1 subunit, containing the RBD, showed reduced fluctuation, while residues in the S2 subunit of the S protein were destabilized (Figure 3A). The most stabilized residues are concentrated near the FABP (Figure 3B), including Phe374, Ser375, Arg408, Thr415, Gly416, and Ile418, with some residues included in the RBD (Gly496, Phe497, Tyr505, and Gln506). When LA binds the FABP, it directly stabilizes interacting residues and, indirectly, additional residues included in the RBD, therefore increasing the frequency of the closed conformation in the population of S proteins. On the other hand, the S2 subunit is destabilized, with multiple residues between Ser659 and Ala1065 with increased fluctuation, particularly residue Asp614, which is critical for the S2 subunit stability.^29^ This stabilization pattern is likely linked to the increased frequency of the S protein in the down conformation in the presence of LA, affecting binding and cell infection.^21^

**Figure 3:**
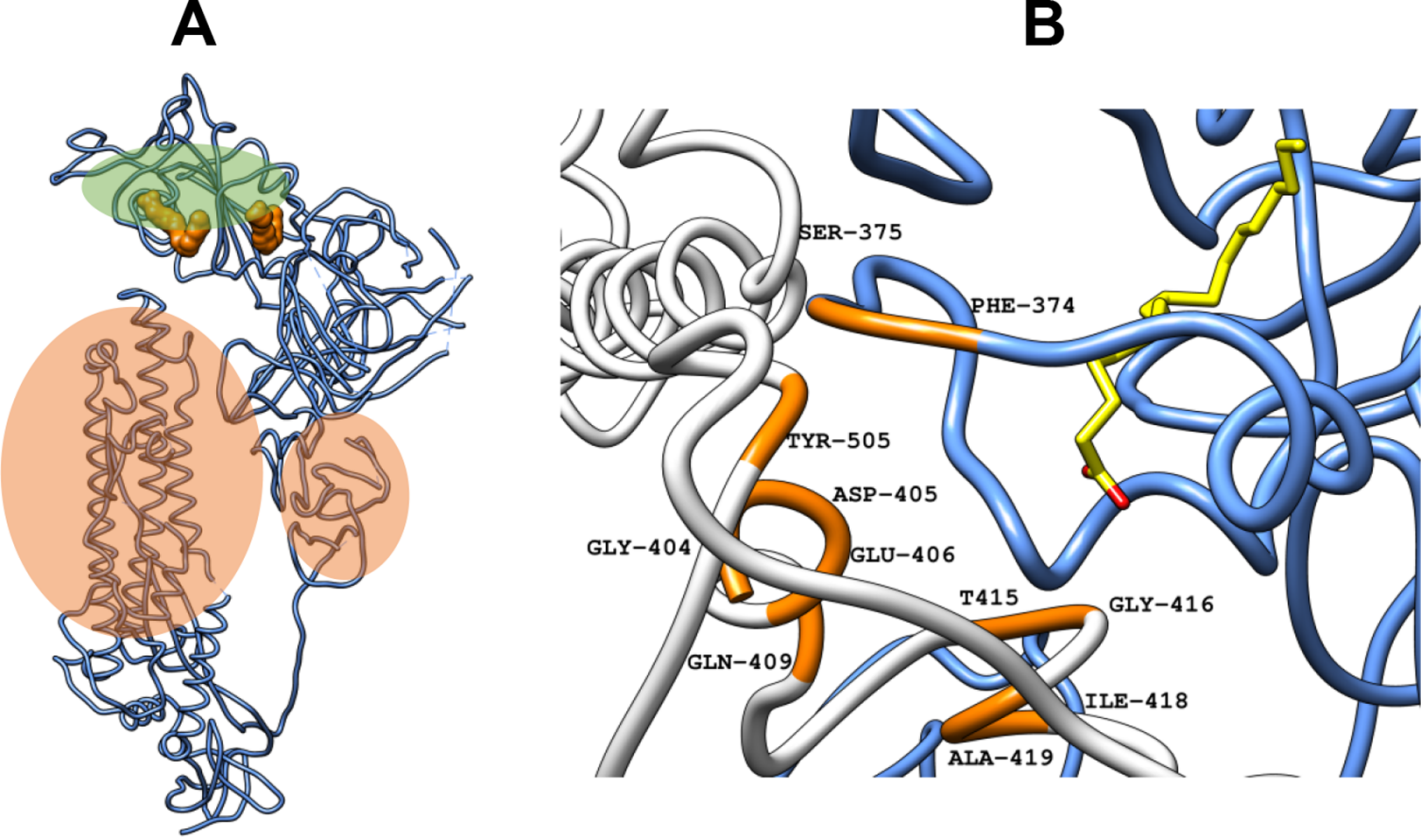
(A) Ribbon representation of a single chain in the trimeric S protein structure (PDB ID 6ZB5). Two FABPs are represented by orange molecular surfaces. Residues highlighted with green circles correspond to reduced fluctuation, while residues highlighted with red circles are linked to increased residue movement. (B) Cocrystallized LA (PDB ID 6ZB5) (carbon atoms in yellow) and FABP in a ribbon representation (blue and white ribbons corresponding to separate RBDs), with the stabilized residues nearby LA (orange ribbon).^30^ Abbreviations: S protein, spike glycoprotein; PDB, Protein Data Bank; FABP, fatty acid binding pocket; LA, linoleic acid.

Additionally, the MOE Site Finder tool was applied to evaluate the stability and availability of FABP for ligand binding across the MD simulations.^31^ This tool detected possible pockets in the S protein, which were matched with the residues forming the FABP (Figure 4).^21^

**Figure 4:**
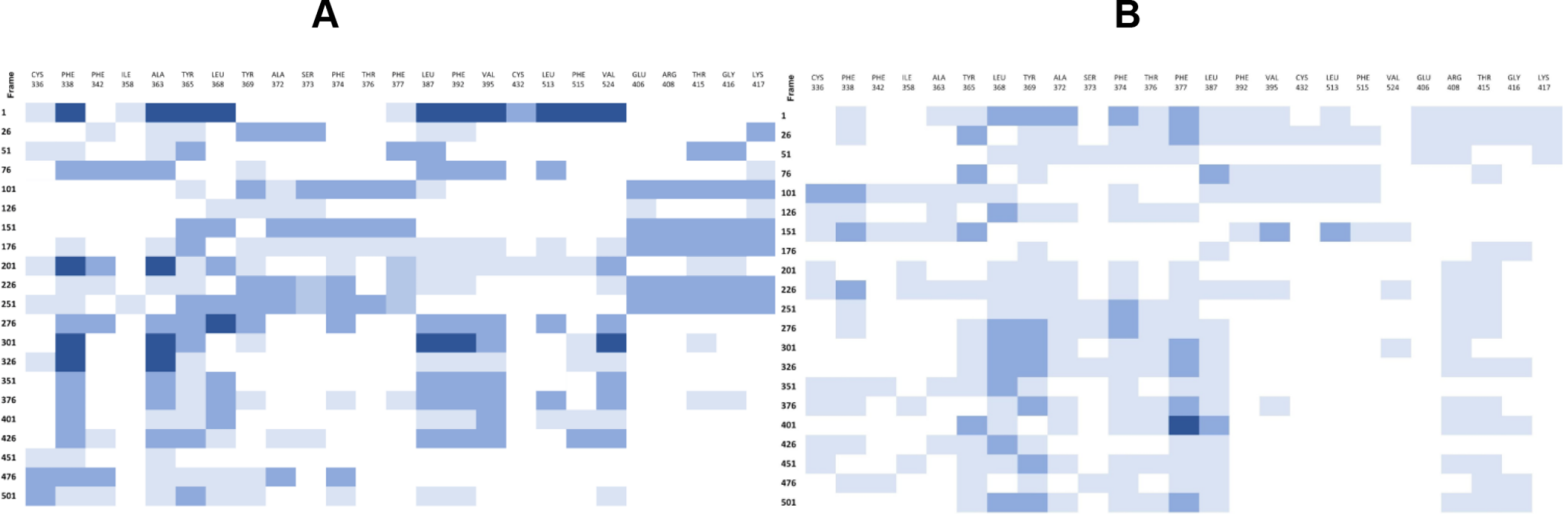
Schematic representation of residues in pockets detected by the Site Finder module in selected frames from MD of the closed conformation (A) and open conformation (B). The color intensity corresponds to detection of each FABP residue in zero, one, two, or three pockets for each frame.^31^ Abbreviations: MD, molecular dynamics; FABP, fatty acid binding pocket.

Overall, the Site Finder analysis revealed that the pocket is accessible across the MD in both the open and closed conformations of the S protein. However, in the closed conformation, the pockets identified in each frame are present in all three spike monomers and contain more FABP residues, suggesting that ligand binding is easier in this state. Despite this, the dynamic nature of the pocket shows available pockets even in an open conformation, whose availability for potential binding varies across the simulation. Nevertheless, the MOE Site Finder always detects a FABP in a spike monomer in every frame. These findings indicate that the FABP is available for binding, in both the open and closed conformations, and that binding stabilizes the S protein, particularly the RBD in the inactive conformation. Importantly, the hydrophilic area is only intermittently detected, although this is the area where the strong electrostatic interactions that stabilize the ligand occur, highlighted during the MD with residues Glu406, Arg408, Thr415, Gly416, and Lys417. However, this analysis showed that the hydrophobic region is critical for initial binding, with residues Cys336, Phe338, Phe342, Ile358, Ala363, Tyr365, Leu368, Tyr369, Ala372, Ser373, Phe374, Thr376, Phe377, Leu387, Phe392, Val395,Cys432, Leu513, and Phe515 maintained during the MD.

Hence, new binding scaffolds should promote more interactions in the hydrophobic area, perhaps exploring the aromatic nature of multiple phenylalanine residues that line the deeper areas of the pocket (Figure 5).

**Figure 5:**
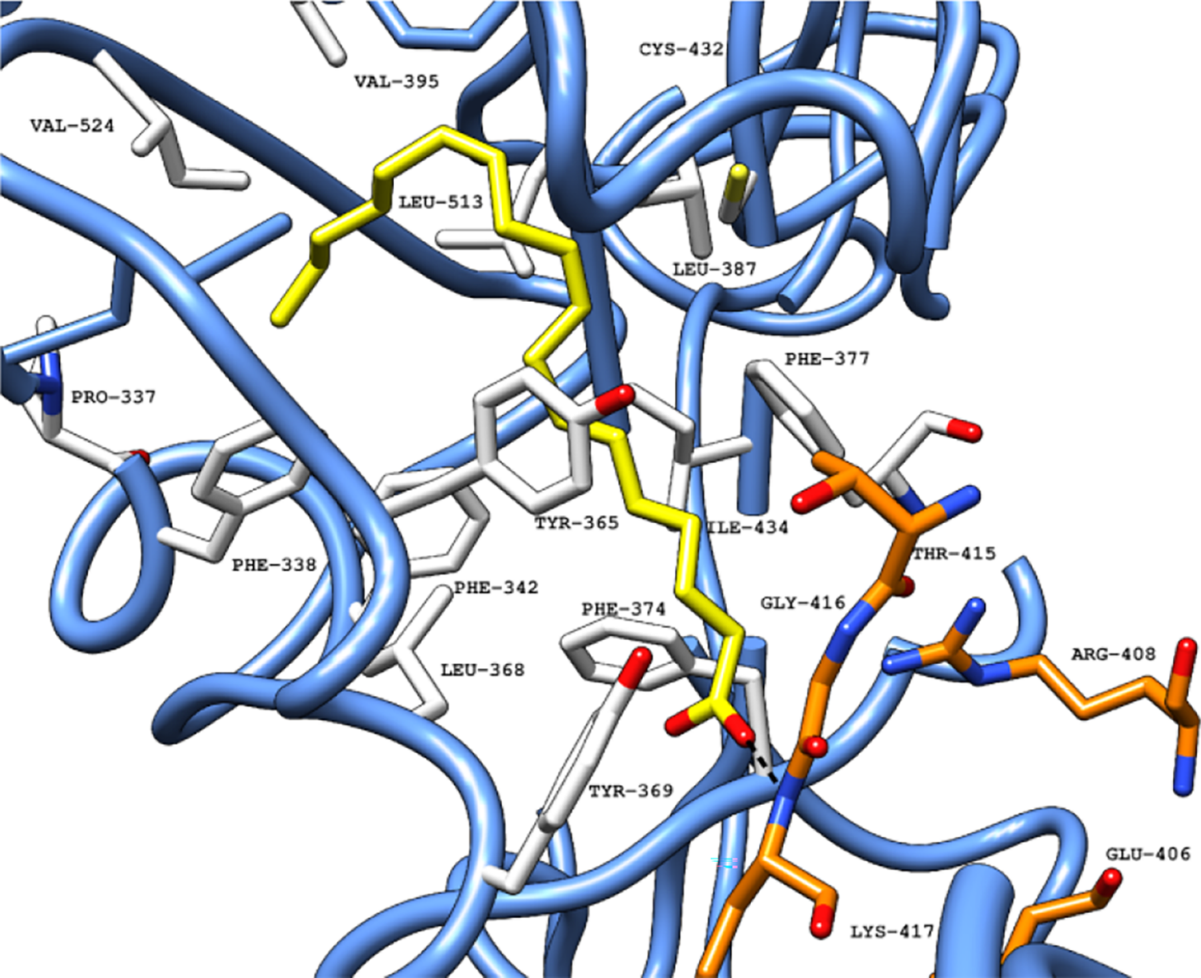
Crystallized LA (PDB ID 6ZB5) (carbon atoms in yellow) in the FABP in a ribbon representation (blue), located between two adjacent RBDs. The pocket is formed by the hydrophobic area (carbon atoms in white) and the hydrophilic area (carbon atoms in orange). Black dashed lines represent polar interactions (hydrogen bonds and electrostatic interactions) between the ligand and amino acid residues in the protein.^31^ Abbreviations: LA, linoleic acid; PDB, Protein Data Bank; RBD, receptor binding domain.

### Docking-Based Virtual Screening

In order to explore the FABP modulatory effects on the spike protein behavior, a docking-based virtual screening was performed, to identify small molecules capable of binding and therefore stabilize an inactive spike conformation. The crystal structure of LA bound to the S protein was used to screen the Enamine Screening Collection library of over 4,000,000 druglike compounds.^32^

The Glide high-throughput virtual screening tool (HTVS)^33^ was employed to virtually screen the database, against each FABP, with the top 50,000 molecules subsequently redocked with Glide Standar Precision (SP).

To avoid potential bias introduced by any single docking program, the docking results (docking poses) were rescored with three scoring functions, Glide Extra Precision (XP), CHEMPLP (PLANTS), and OpenEye (ScorePose).^34–36^ After applying an in-house optimized consensus scoring procedure, 3000 molecules for each binding site were chosen for visual inspection.^37^ Combining the top molecules with the best predicted interactions in the three pockets, along with analysis of druglike properties, resulted in a final selection of 18 molecules (Figure 6), which were purchased from Enamine and evaluated in binding assays and cell-based antiviral assays. As an example, the predicted binding for compound **1** is superimposed with LA in its binding site in Figure 7.

**Figure 6:**
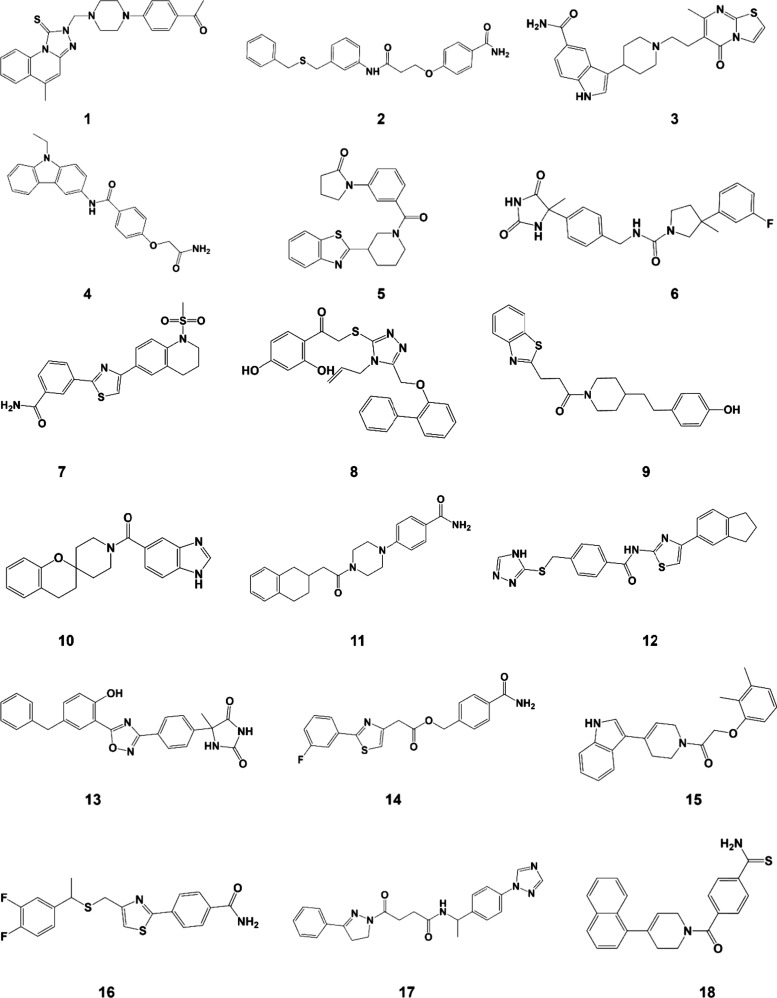
Chemical structures of compounds selected after the structure-based virtual screening and purchased from Enamine.

**Figure 7:**
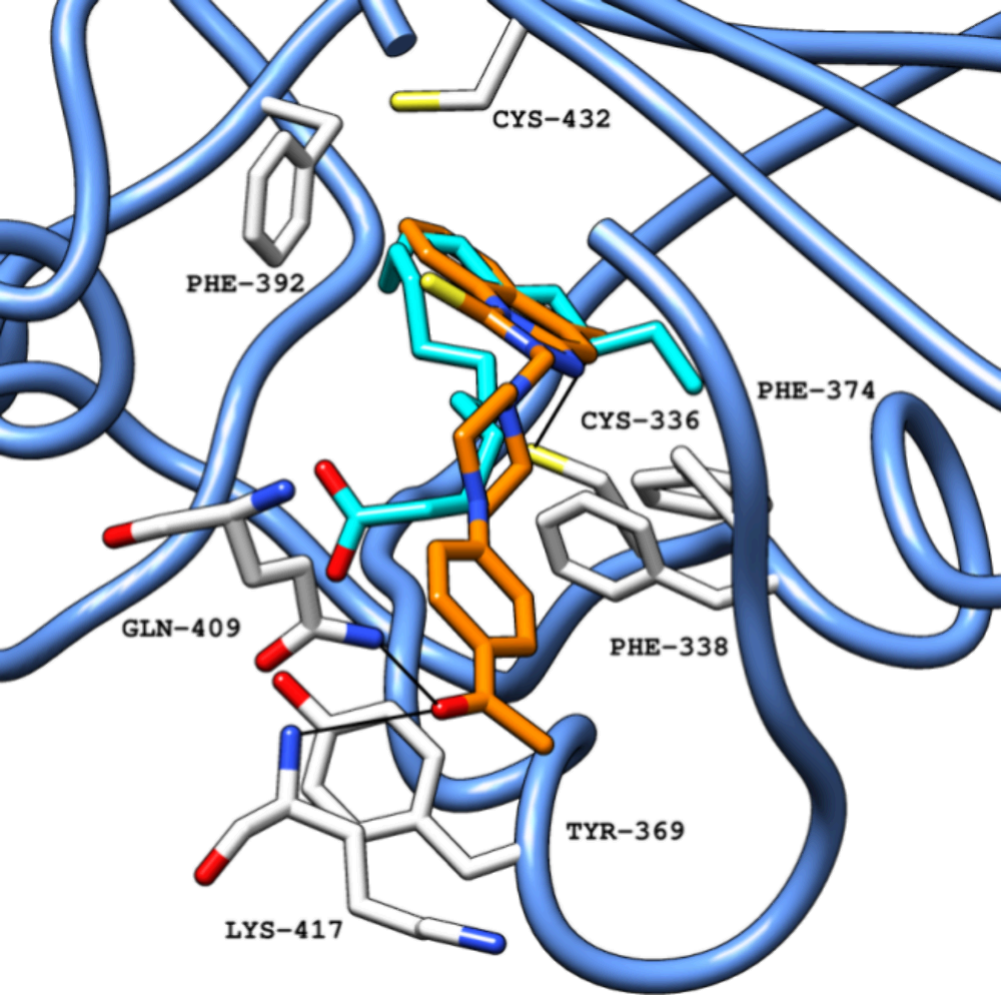
Crystallized LA (PDB ID 6ZB5) (carbon atoms in light blue) superimposed with docking pose for compound **1** (carbon atoms in orange) in the FABP in a ribbon representation (blue), obtained with Glide SP. Black lines represent polar interactions (hydrogen bonds and electrostatic interactions) between the ligand and amino acid residues in the protein.^30^ Abbreviations: LA, linoleic acid; PDB, Protein Data Bank; FABP, fatty acid binding pocket.

Overall, the 18 selected compounds were predicted to achieve good pocket occupation and interactions with buried hydrophobic residues along with multiple electrostatic and H-bond interactions with hydrophilic residues in the pocket entrance entrance. The main difference when compared with LA was the frequent presence of aromatic groups, against alkane chains in LA. The presence of aromatic features is shared with previously identified FABP bioactive molecules, such as lifitegrast, retinoids, or SPC-14, with our molecular dynamics studies also pointing to possible stronger binding by exploring the hydrophobic area of the pocket. When compared with LA, compound **1** also extended further to the hydrophilic area and established direct contacts with the neighboring residues. Other selected compounds showed variability in pocket occupation with varying focus in interactions with either hydrophobic or hydrophilic residues.

### Biological Assays

#### Binding Assays

An ELISA-based inhibition assay was used to assess whether the compounds selected inhibit the S-ACE2 interaction. The compounds were tested at 200 μM, and their activity was compared with positive and negative controls (vehicle, 2% dimethyl sulfoxide (DMSO)). LA was selected as a positive control due to its established inhibitory effect on the S-ACE2 interaction, showing 100% inhibition of the RBD-ACE2 binding at a concentration of 8.9 mM in *in vitro* assays.^18^ Additionally, palmitoylethanolamide (PEA) was also used as a positive control, as it has been confirmed to reduce RBD binding with ACE2 by ∼50%.^17^ At 200 μM, six compounds had stronger inhibitory activity than PEA (17% inhibition), but none reached LA inhibition (84% inhibition; Figure 8).

**Figure 8:**
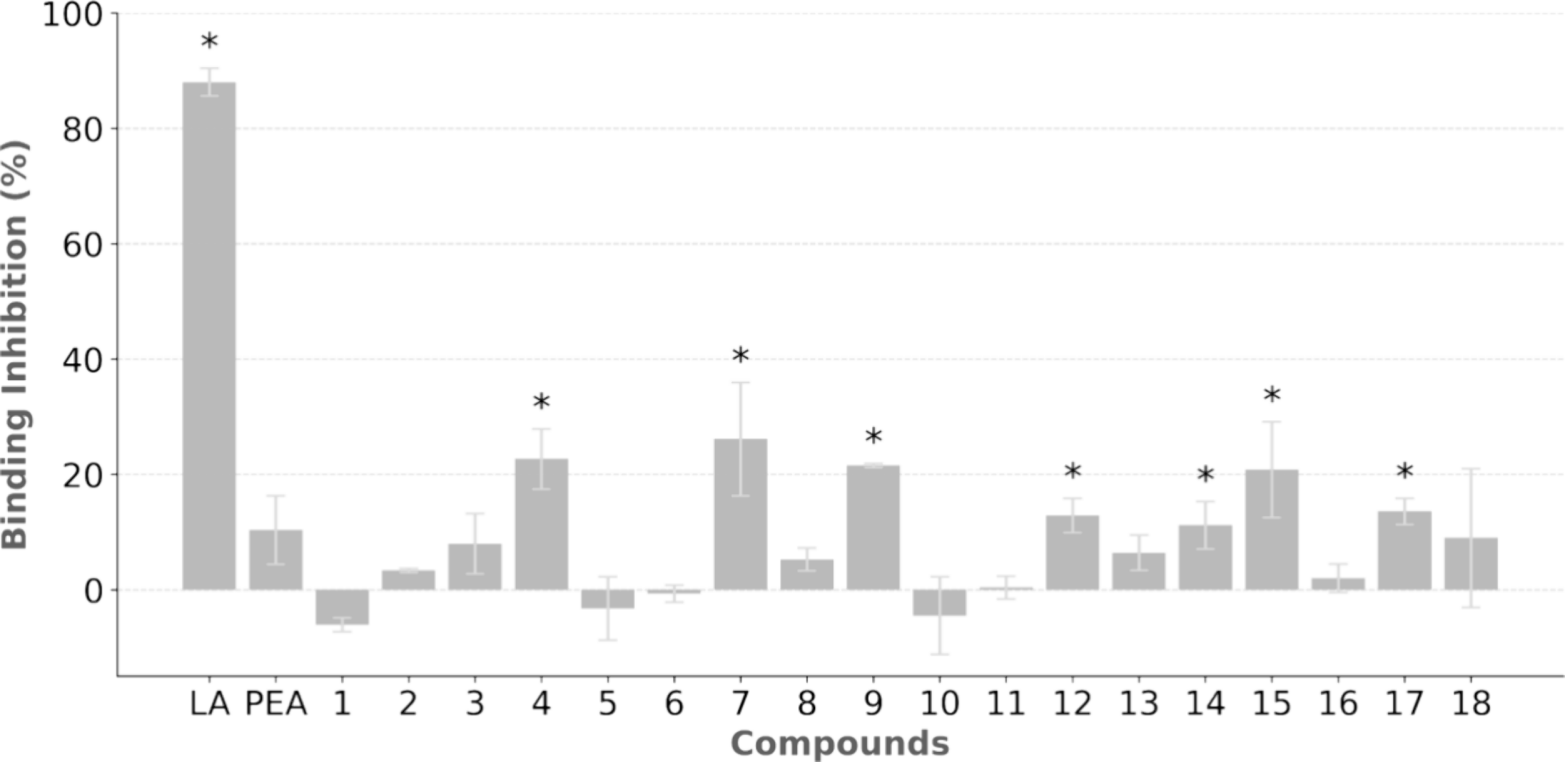
Inhibitory activity of binding assays for the S-ACE2 interaction for the screened compounds in the *X*-axis, tested at 100 μM. Inhibition in the *Y*-axis was determined as a percentage of the vehicle-treated cells (2% DMSO). Bars represent the mean ± SEM from three experimental repeats. *Compounds with higher activity than PEA. Abbreviations: S-ACE2, spike glycoprotein-angiotensin converting enzyme; DMSO, dimethyl sulfoxide; LA, linoleic acid; PEA, palmitoylethanolamide.

The maximum detected inhibition was 28% for compound **4**, with compounds **7**, **9**, and **15** reaching 20% inhibition of the interaction, while compounds **12** and **17** also surpassed PEA. When compared with linoleic acid at 200 μM, reaching 84% inhibition of interaction-derived signal, these compounds have shown a reduced ability to affect the interaction. However, since PEA can affect the virus–host interaction in virion-based assays, the screened compounds still hold the potential to replicate this effect.

### Cell-Based Infection Assays

The antiviral activity of the screened compounds was evaluated in Vero E6 cells in cell-based SARS-CoV-2 infection assays with the original Wuhan strain. The compounds were evaluated for their ability to protect cells from virus-induced cytopathic effects (CPE), caused by SARS-CoV-2 damages to the host cell and observed in Vero E6 cells by microscopy.^39^ To quantitatively assess CPE, the CellTiter-Glo methodology was used to measure the amount of ATP present in the medium since CPE leads to ATP release and degradation.^39^ The screened compounds were tested at multiple concentrations, with a 0.03–200 μM range (Figure 9). The compound with the highest activity also showed a dose–response curve (Figure 10A). However, inhibition of CPE does not provide information on the number of infectious virions within a sample; hence, a TCID_50_ titration assay (median tissue culture infectious dose) was performed to assess the amount of replication-capable lytic virions released (Figure 10B).

**Figure 9:**
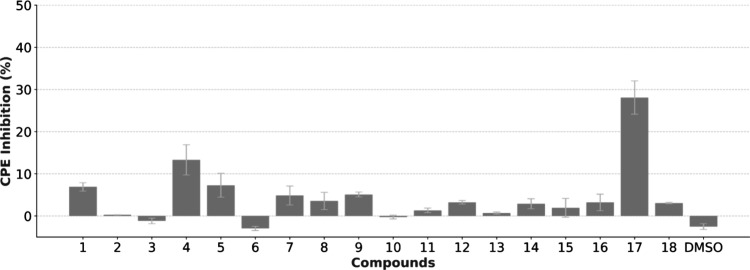
Inhibitory activity of viral induced CPE by the screened compounds in the *X*-axis, at 67 μM, after 72 h of infection, in Vero E6 cells, measured using the CellTiter-Glo method. CPE inhibition in the *Y*-axis was determined by comparing cells exposed to compounds with cells infected in the absence of compounds. The bars represent the mean ± SEM from three experimental repeats. Abbreviation: CPE, cytopathic effect.

**Figure 10:**
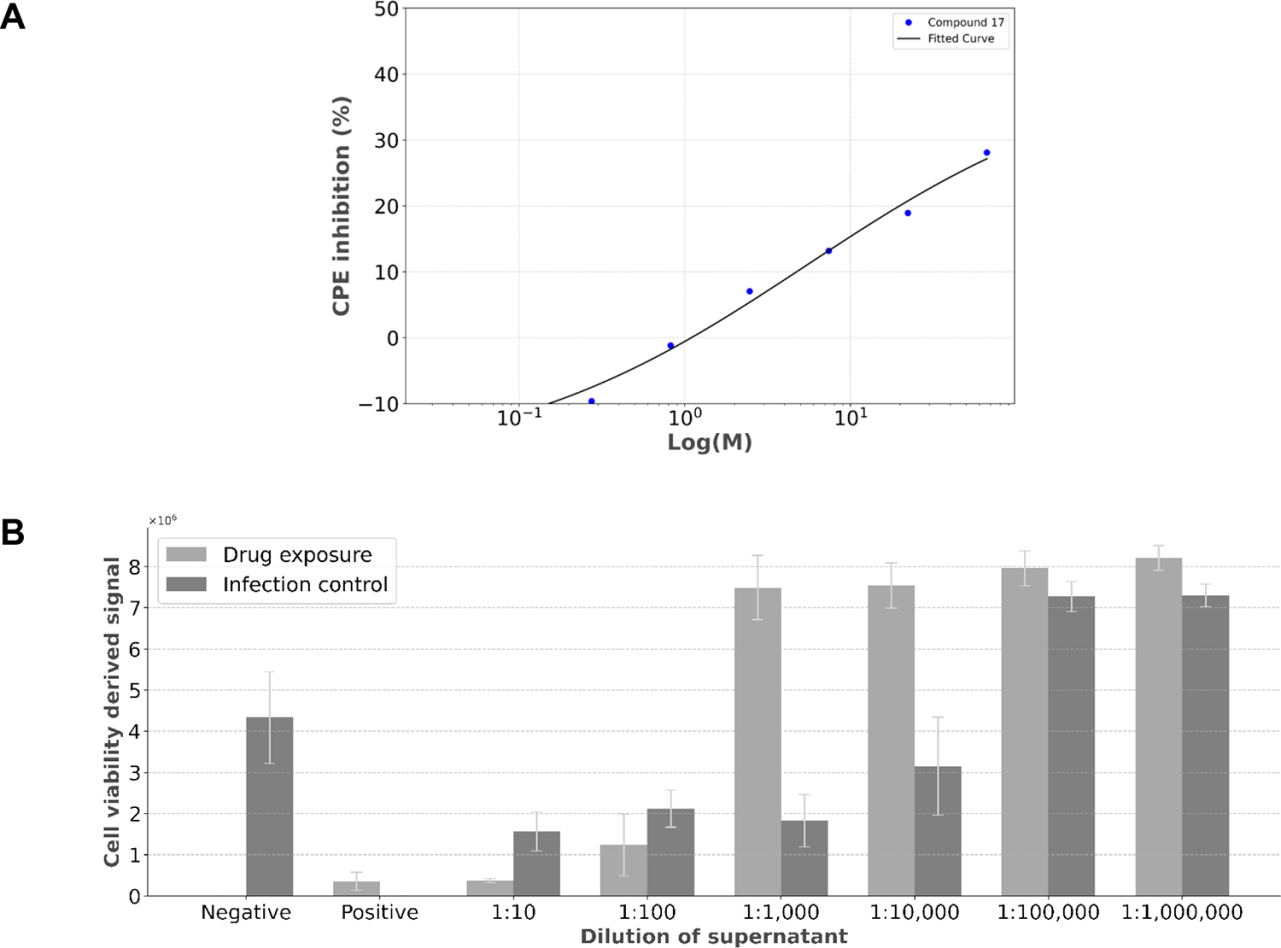
(A) Dose–response curve for compound **17** in Vero E6 cells, corresponding to the inhibitory activity of SARS-CoV-2-induced CPE in the *Y*-axis. The *X*-axis represents compound concentration expressed on a logarithmic scale. (B) Titration assay for compound **17** based on cell viability in the *Y*-axis measured with the CellTiter-Glo method, for each dilution of the viral supernatant in the *X*-axis. The bars represent the mean ± SEM from three experimental repeats. Abbreviation: CPE, cytopathic effect.

Among the compounds screened in this assay, compound **17** showed the strongest antiviral activity at every concentration, with a maximum 28% reduction in viral induced CPE at 67 μM and activity over 10% at 2.5 μM. Overall, cytotoxicity was a limiting factor at higher concentrations. At 67, μM compound **4** also surpassed 10%, while at 22 μM, where cytotoxicity is less relevant, compounds **1** and **15** also achieved 10% inhibition.

A dose response curve was obtained for compound **17**, with strong cytotoxicity consistently observed at 200 μM, which likely limits higher activity. The titration procedure was applied with the highest active concentration (67 μM). While cell viability at early dilutions was similar in the presence and absence of compound **17**, the drug-exposed group showed cells with regular growth two 10-fold dilutions before the control. These results showed that the presence of compound **17** during the initial infection not only inhibited CPE but also resulted in a decrease of 81% in the number of infectious virions released.^40^ Besides the quantifiable effect, the reduction in CPE for compound **17** was visible by microscopy, as healthy cells were observed. Overall, compound **17** was capable of partial inhibition of CPE during viral infection, while reducing the number of infecting new virions released, demonstrating the ability to inhibit viral induced effects.

### Predicted Binding of Antiviral Hits

Four compounds showed an increased ability to inhibit viral induced CPE (compounds **1**, **4**, **5**, and **17**), with their predicted binding pattern obtained from the docking studies. Following the observations for LA, extensive interactions are predicted in the hydrophilic portion of the pocket, although multiple aromatic rings extend toward the hydrophobic area, which is predicted to provide a better occupation and establish aromatic interactions (Figure 11).

**Figure 11:**
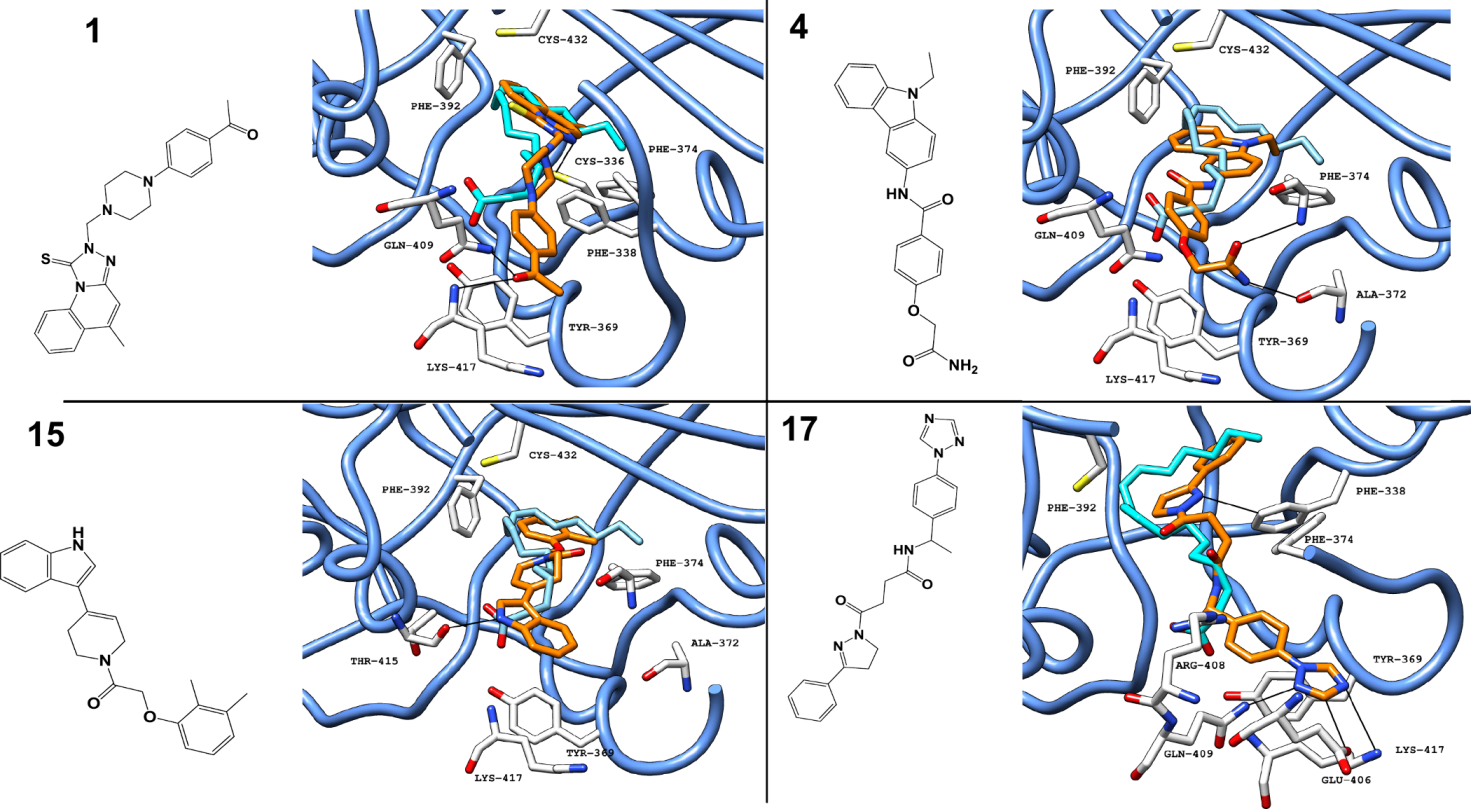
Crystallized LA (PDB ID 6ZB5) (carbon atoms in cyan) superimposed with LA in the FABP in a ribbon representation (blue ribbon), along with the predicted binding pose (obtained with Glide SP) for small-molecule inhibitors with the highest antiviral activity (compounds **1**, **4**, **15**, and **17**) and with the chemical structure. Black lines represent polar interactions (hydrogen bonds and electrostatic interactions) between the ligand and amino acid residues in the protein.^30^ Abbreviations: LA, linoleic acid; FABP, fatty acid binding pocket.

In the four compounds with the strongest activity, the aromatic rings are a recurring feature, with phenylalanine residues (Phe338, Phe374, and Phe392) consistently predicted to establish π–H and π stacking interactions. On the other hand, regarding the hydrophilic entry of the pocket, compounds **1**, **4**, and **17** have hydrophilic moieties (ketone, amide, and triazole, respectively), while compound **15** exposes an indole moiety and therefore is predicted to rely more upon hydrophobic interactions, with only one H-bond predicted. On the other hand, the ketone, amide, and triazole groups are predicted to establish extensive interactions, with multiple residues in the pocket entrance (electrostatic interactions including H-bonds). Additionally, compounds **1** and **15** show a less efficient pocket occupation, even compared to LA, which extends more into the pocket.

Regarding the most active antiviral, compound **17**, the binding pattern can be compared to that of LA, having two main differences. Despite a similar extension in the hydrophobic area, compound **17** is predicted to achieve better pocket occupation due to the aromatic nature of the scaffold, particularly in the vicinity of buried phenylalanine residues, allowing π stacking and π–H interactions, with additional π–H interactions predicted with Cys432 and Tyr369 in the middle area of the pocket. In addition to a good occupation of the deeper pocket areas, compound **17** also extends toward functional groups in hydrophilic residues in the entrance of the pocket. The occupation of the anchoring entrance with a triazole group results in strong H-bond interactions with Glu406, Glu409, and Lys417 and additional π–H interactions with Arg408, which strongly stabilize the molecule in the pocket.

### Cell Viability

A preliminary cytotoxicity evaluation before cell infection for the 18 compounds was performed using Vero E6 cells, kidney epithelial cells originally isolated from African green monkey (*Chlorocebus* sp.).^41^ This is a commonly used cell model for coronavirus infection as it highly expresses ACE2, the functional receptor recognized by the S protein, and shows characteristic CPE.^42^ In the virtual screening selection protocol, predicted toxicity was an important consideration, with ADME and PAINS (pan-assay interference compounds) analysis included to exclude potential toxic compounds (Supporting Information, Table S1).^38^

In addition to the test compounds, cells were treated with DMSO since it is capable of altering cell membrane permeability and selectivity, justifying its use as a control for cytotoxicity.^43^

In the preliminary assay control, cell growth for most compounds was observed in the initial stage, while stabilization in cell viability is generally observed, with DMSO exposed cells showing strong cytotoxicity. Overall, a concentration over 100 μM could be used in infection assays, to allow maximum inhibitory effects without high levels of toxicity (Supporting Information, Figure S1). During viral infection assays, concentration causing 50% cytotoxicity (CC_50_) was determined for all screened compounds (Supporting Information, Figure S2), with CC_50_ values ranging from 59 to 220 μM, and strong cytotoxicity only at high concentrations. The most active compound (**17**) showed a CC_50_ of 90 μM. The preliminary evaluation of cytotoxicity was, therefore, critical to guarantee adequate separation between activity and cytotoxicity at testing concentrations.

Overall, from an initial virtual screening study targeted at the S protein, compound **17** was identified as the most active compound, reducing viral induced CPE by around 30%, along with a reduction in the number of viral particles released. It also showed an approximately 20% ability to reduce the S protein-ACE2 interaction in binding assays. Therefore, it is likely that the activity shown in the infection assays is, at least in part, derived from an effect on the FABP, stabilizing the inactive conformation of the RBD, which becomes incapable of recognizing target receptors and infecting new cells. Nevertheless, this action through the FABP has not been directly confirmed, requiring further experiments such as surface plasmon resonance, as observed for lifitegrast, or structural determination with cryo-EM, as observed for linoleic acid.

### Analogue Identification

Starting from the compound with the highest activity, with compound **17** showing micromolar activity, a few commercial analogues were selected by similarity, for activity confirmation and the study of structure, activity relationships. The 20 selected analogues were divided in three main groups: (1) substitution of the terminal triazole by apolar groups, such as tertbutyl, phenyl or methyl; (2) substitution of the terminal triazole by functional polar entities such as tetrazole, thiazole and amide; (3) substitution of the linker between the aromatic groups, extension or reduction in overall size, and new scaffolds (Figure 12). These changes were intended to explore the interactions with the hydrophilic anchor in the pocket, improving pocket occupation and exploring new interactions, as well as improving druglike properties.

**Figure 12:**
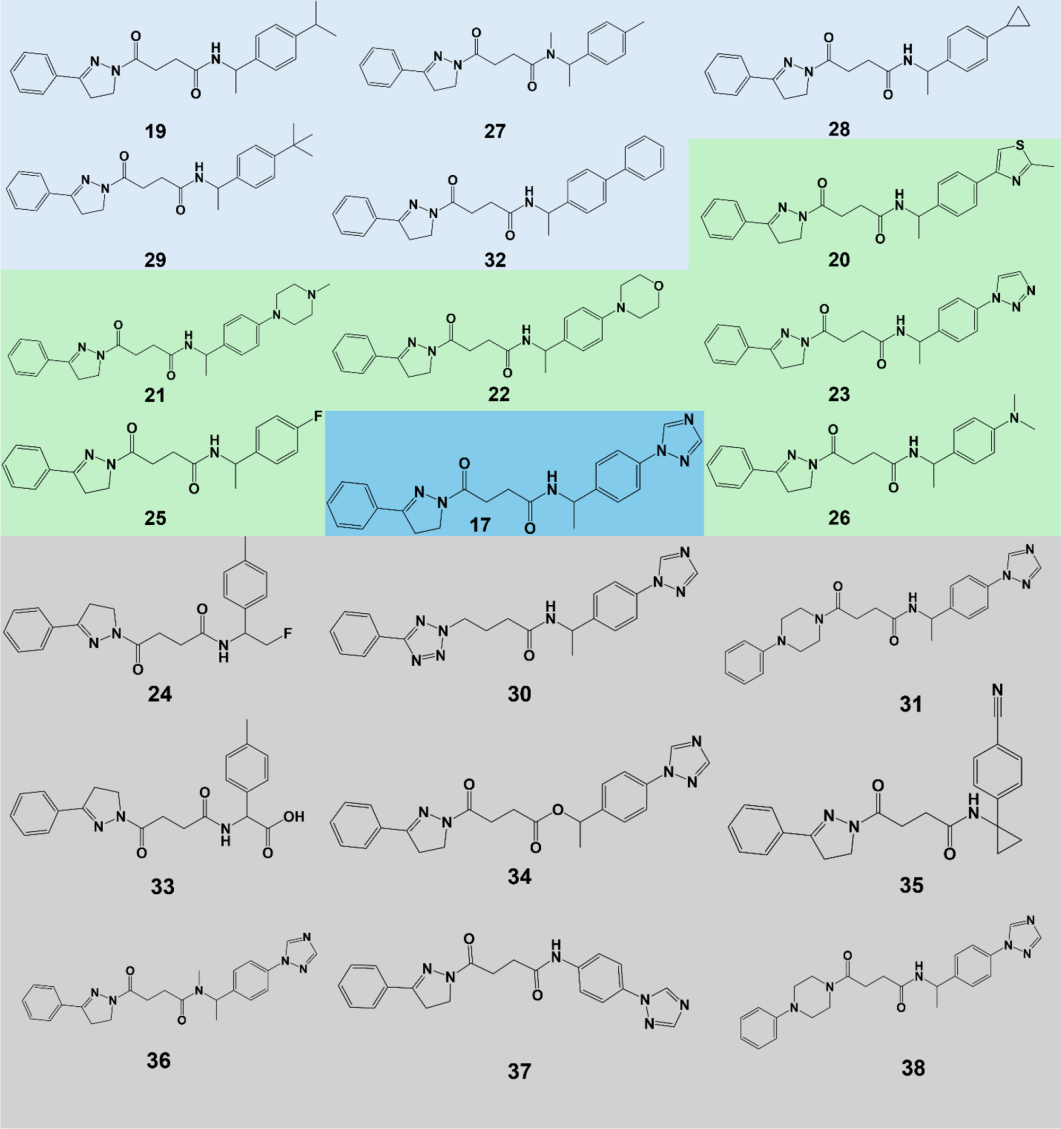
Chemical structures of analogue compounds to the strongest antiviral identified, divided in three categories and the original bioactive compound, with hydrophobic substitutions (light blue), hydrophilic substitutions (green), other changes (gray), and the original compound (blue).

### Biological Evaluation of the Hit Analogues

The 20 structural analogues selected (compounds **19**–**38**) and the parent compound (compound **17**), used as a positive control, were evaluated for their ability to inhibit the viral induced CPE and viral replication. The compounds were tested at concentrations ranging from 0.15 to 100 μM. Since compound **17** was able to reduce CPE evident by microscopy, only compounds with the ability to visibly reduce CPE were subject to quantitative testing, upon confirmation of positive and negative controls. Upon optical confirmation of CPE, 10 compounds were subject to quantitative evaluation of activity and toxicity, with CPE inhibition at 33 μM showing maximum activity levels (Figure 13A), while cytotoxicity was limited with CC_50_ values ranging from 37 to 243 μM and 50% cytotoxicity not detected at 100 μM (Supporting Information, Figure S3). The best analogue, compound **36**, exhibited a maximum of 91% inhibition of CPE at 33 μM and a dose–response curve (Figure 13B).

**Figure 13:**
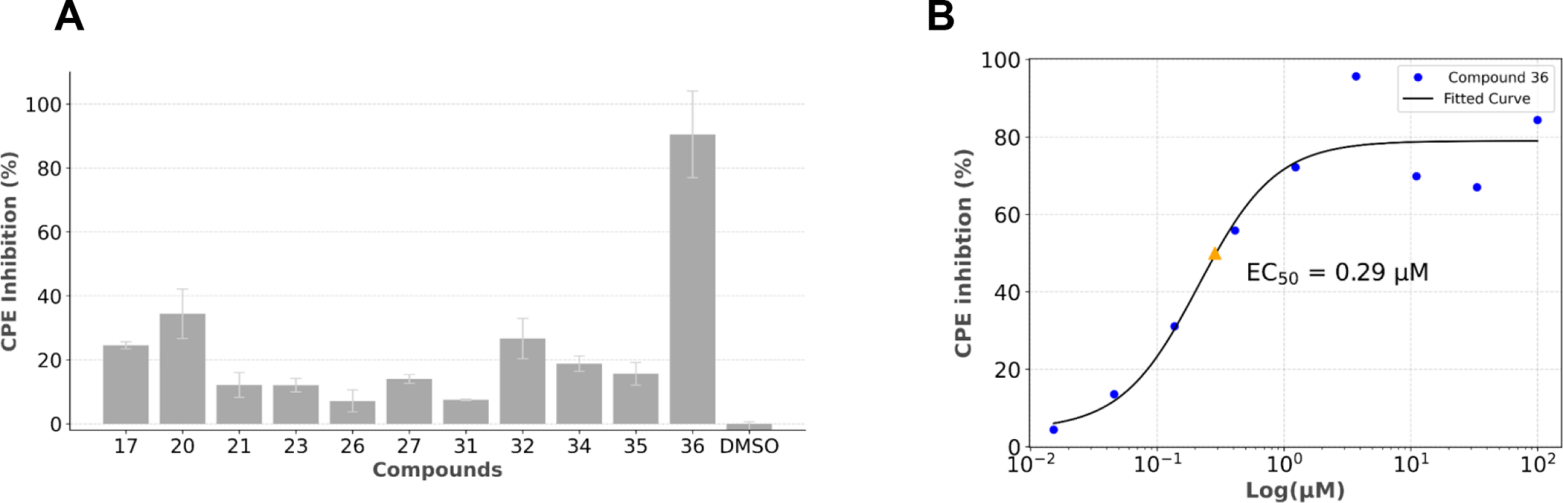
(A) Inhibitory activity of viral induced CPE by analogue compounds in the *X*-axis at 33 μM, after 72 h of infection, in Vero E6 cells, measured using the CellTiter-Glo method. The *Y*-axis represents CPE inhibition, determined by comparing cells exposed to compounds to cells infected in the absence of compounds. The bars represent the mean ± SEM from three experimental repeats. (B) Dose–response curve for compound **36** in Vero E6 cells, corresponding to the inhibitory activity of SARS-CoV-2-induced CPE in the *Y*-axis. The *X*-axis represents compound concentration expressed on a logarithmic scale. Abbreviations: CPE, cytopathic effect; DMSO, dimethyl sulfoxide; EC_50_, half-maximal effective concentration.

From the analogues that induced a visible reduction by microscopy in viral induced CPE, most had their activity confirmed in the infection assays, with nine showing at least 10% inhibition at 33 μM and compound **36** showing 91% inhibition with an EC_50_ of 0.29 μM. When compared with compound **17** (23%), compound **20** (34%), compound **32** (27%), and compound **36** (91%) achieved stronger inhibition of viral induced CPE. Overall, cytotoxicity was limited, not reaching 50% reduction in cell viability in all compounds, apart from compounds **27** and **34**.

### Structure–Activity Analysis

The analogues selected for the optimization stage of this study introduced multiple structural modifications aimed at increasing activity and establishing a preliminary structure activity relationship. For the hydrophobic substitution analogues, the introduction of an aliphatic chain in the hydrophilic anchor is associated with a loss of activity, with interactions in this area severely reduced (Figure 14).

**Figure 14:**
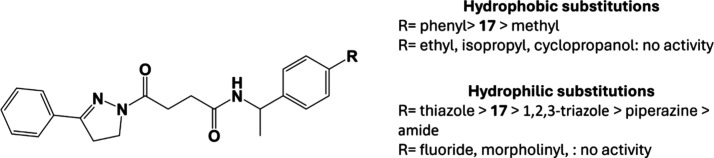
Quantitative/qualitative structure–activity relationship concerning the first and second groups of analogues.

The aromatic nature of the triazole is confirmed as critical, since compound **32** (phenyl) resulted in increased activity, while the importance of interaction in the hydrophobic area of the pocket is highlighted by the activity maintained in compound **27** (methyl). Regarding the hydrophilic substitutions (Figure 15), the increased activity of the methylthiazole substitution (compound **20**) could be due to increased aromatic and hydrophilic interactions, or potentially by an increase in LogP (2.36 (compound **17**) and 3.91 (compound **20**)), which, given the hydrophobic nature of the FABP, might benefit initial binding and therefore activity.

**Figure 15:**
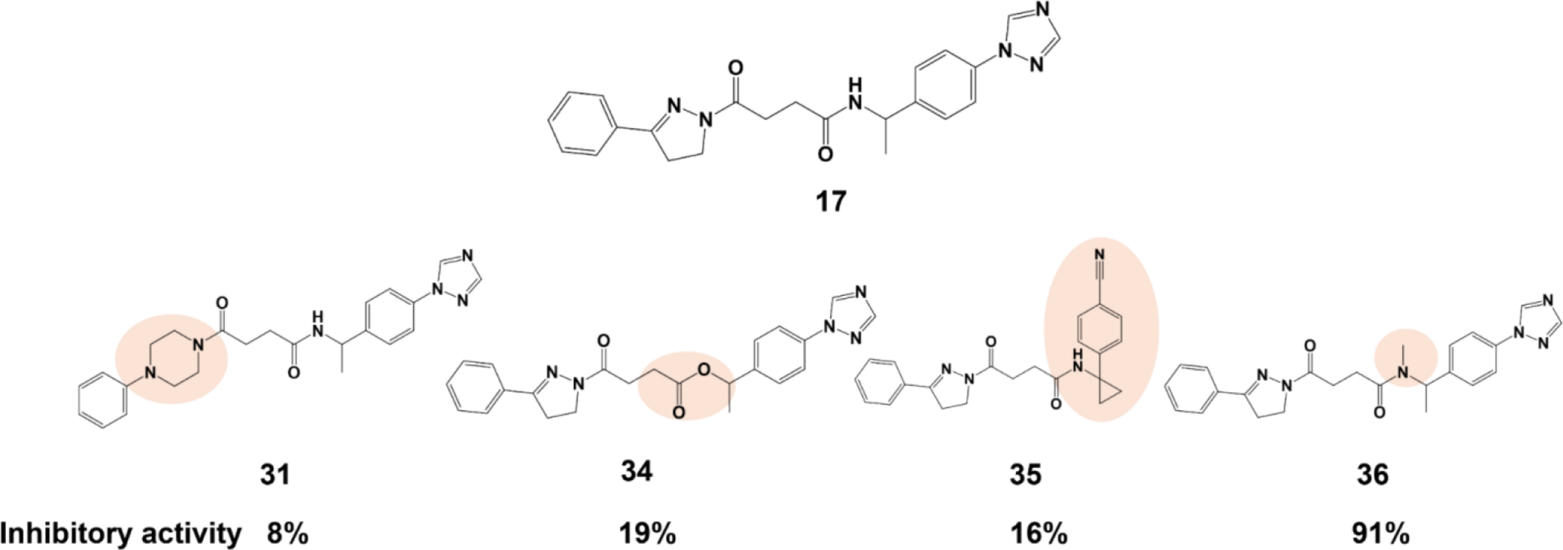
Chemical structures of analogue compounds from the third category that resulted in compounds with inhibitory activity.

On the third set of analogues, the strongest antiviral analogue (compound **36**) represented a significant improvement when compared with compound **17**, although they only differ in the methylation of a linking secondary amine group, resulting in a tertiary amine (Figure 15). The nitrile group in compound **35** might also be an interesting terminal group since some activity is retained.

Although compound **36** loses the ability to be a hydrogen donor for H-bonds, the ability to be a receptor for H-bonds, such as with Tyr365 and Tyr369, is reinforced, which might result in a stronger anchoring effect of this portion of the molecule (Figure 16). Additionally, the increase in LogP (2.36 (compound 1**7**) to 2.62 (compound **36**)) might also improve the initial interaction with the target.

**Figure 16:**
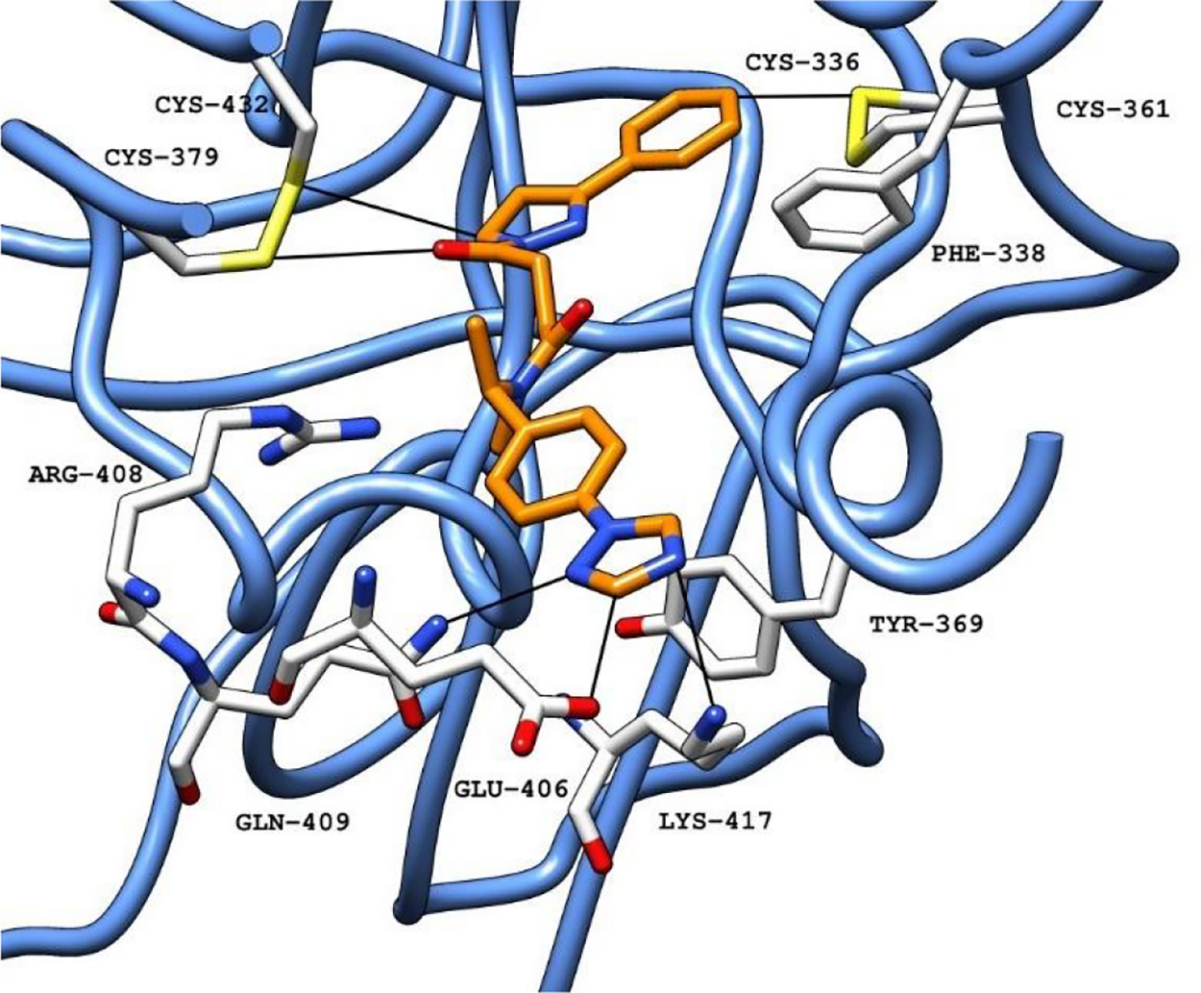
Predicted binding pose for compound **36** (carbon atoms in orange) obtained with Glide SP, in the FABP in a ribbon representation (blue) (PDB ID 6ZB5). Black lines represent polar interactions (hydrogen bonds and electrostatic interactions) between the ligand and amino acid residues in the protein.^30^ Abbreviations: FABP, fatty acid binding pocket; PDB, Protein Data Bank.

### Activity against SARS-CoV-2 Variants of Concern

In addition to the Wuhan SARS-CoV-2 strain, compound **36** was also tested against the SARS-CoV-2 Delta and Omicron variants of concern.^10,23^ Since the FABP is not under selective pressure from antibodies and vaccine-induced immune response, it is expected that active compounds maintain the ability to inhibit cell-induced CPE and affect the virus life cycle.^22^ Therefore, compound **36** was tested against the SARS-CoV-2 Delta and Omicron variants. The results with the Delta variant followed a similar pattern to the Wuhan variant, with a maximum inhibition detected at 11 μM, while a dose response is present toward lower dilutions (Figure 17). Compound **36** showed a maximum inhibition of CPE by 78% at 33 μM and an EC_50_ of 5.77 μM.

**Figure 17:**
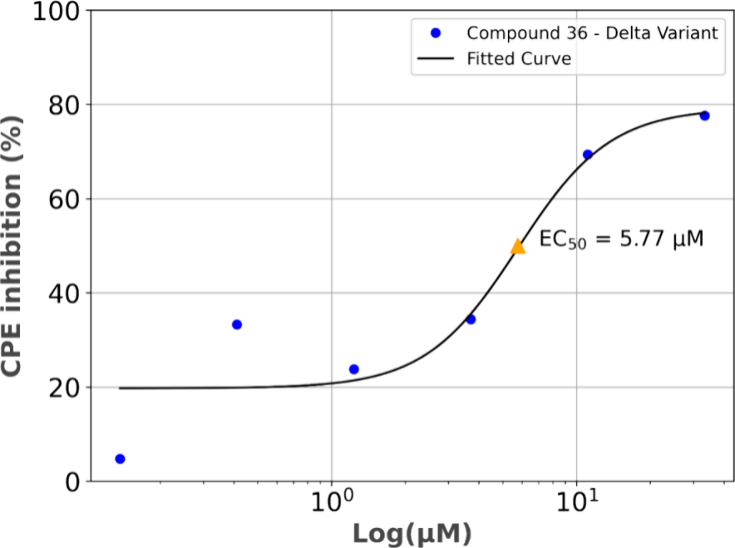
Dose–response curve for compound **36** in Vero E6 cells, corresponding to the inhibitory activity of SARS-CoV-2 Delta variant-induced CPE in the *Y*-axis. The *X*-axis represents compound concentration expressed on a logarithmic scale to better visualize the dose–response relationship. Abbreviation: CPE, cytopathic effect.

An important deviation from expected behavior with the Delta strain was detected when compared with the Wuhan strain. Infected cells did not show CPE after 96 h and only after an additional 72 h period could CPE be observed. Further assays performed with the Omicron strain did not show CPE even after this extended period (Supporting Information, Figure S4). There seems to be a link between viral evolution from the initial strains (Wuhan and Alpha) to later strains (Delta and Omicron) that leads to reduced CPE.^44^ Cell entry by the SARS-CoV-2 Wuhan strain can proceed by two ways, S protein initiated cell fusion after a proteolysis by transmembrane protease serine 2 (TMPRSS2) or alternatively by endocytosis, with the acidic pH activating cathepsin for the proteolysis step.^45^ The Omicron variant has a strong preference for the endocytosis pathway, whereas the Wuhan and Delta strains preferentially initiate entry by the TMPRSS2 cleaved S protein. Viral induced CPE is dependent on infection through adjacent cells, which requires TMPRSS2-mediated activation. This change is also associated with a variation in cell tropism: early SARS-CoV-2 strains target the lower respiratory tract, rich in TMPRSS2, while Omicron targets the upper respiratory tract, rich in cathepsin, which justifies the lack of observed CPE for the Omicron variant.^39,45^

Compound **36** maintained activity in the heavily mutated Delta variant, showing conservation of the FABP, which is also conserved in other human CoVs, particularly in the other highly pathogenic CoVs: SARS-CoV and MERS-CoV.^23^ Therefore, it would be important to perform further assessment of pan-coronavirus activity, as compound **36** holds the potential for broad-spectrum activity. Overall, the activity shown by this molecule could be highly relevant not only to the current pandemic but also to potential future emerging new CoVs.

### RT-ddPCR

SARS-CoV-2-infected cells were used for antiviral assays, with the resulting viral induced CPE analyzed. To confirm and quantify that the pathogen causing this effect indeed was SARS-CoV-2, PCR analyses were performed on the supernatant retrieved from the infected cells. To this end, we applied reverse transcriptase (RT) digital droplet (dd)­PCR.^46^ For this PCR, the target samples are compartmentalized in thousands of droplets together with the PCR reagents, making multiple reactions that occur simultaneously.^46,47^ Due to this, absolute quantification is possible.^47^ The supernatant samples from cells infected with SARS-CoV-2 Wuhan strain and treated with compound **36** were retrieved and analyzed by RT-ddPCR (Table 1).

**Table 1:** ddPCR Results, with Detected Viral Genomic Copies for SARS-CoV-2-Infected Cells Treated with Compound **36** Compared to Nontreated and Infected Cells

compound	copies/20 μL
**36**	16.9
positive control	146.6

The number of viral genome copies present in the supernatant after infection was reduced by 89% at 33 μM. Additionally, TCID_50_ was also determined for compound **36** (Table 2), with a reduction of 90% of replication-capable lytic virions. Hence, both independent methodologies point to a similar reduction in infectious virions released during infection, with compound **36** capable of significantly affecting the virus life cycle.

**Table 2:** Titration Results for Infection with the SARS-CoV-2 Wuhan Strain Exposed to Compound **36**

TCID_50_/mL	drug	no drug	PFU/mL	drug	no drug
compound **36**	3.6	4.6		2.49 × 10^7^	1.25 × 10^8^

## Conclusions

The FABP shows great promise in modulation of S protein behavior and, consequently, SARS-CoV-2 infection due to its essential role in cell recognition and entry. However, none of the currently approved SARS-CoV-2 treatments target the S protein or the FABP. Given the conservation of the pocket across highly pathogenic human coronaviruses, it holds the potential for pan-coronavirus activity, which is crucial for addressing both current and future coronavirus outbreaks.^23^

In this work, the FABP effects on the S protein behavior were explored using molecular dynamics simulations, which guided a docking-based virtual screening of a commercial library of small-molecule, druglike compounds. This approach led to the identification of potential inhibitors, which were purchased and evaluated in direct binding and cell-based antiviral assays. Among these, one compound demonstrated significant inhibition of viral-induced effects, likely by binding to FABP, thus validating the computational approach. This compound served as the starting point for evaluating 20 structural analogues, leading to the identification of a submicromolar inhibitor of SARS-CoV-2 with low toxicity. These findings might provide a promising starting point for further optimization and drug development efforts against SARS-CoV-2.

## Materials and Methods

### Molecular Modeling Studies

#### Molecular Dynamics Simulations

Molecular dynamics and molecular docking studies supporting the virtual screening study were performed on an Asus WS X299 PRO Intel i9, 10980XE CPU @ 3.00 GHz × 36 running Ubuntu 18.04 (graphic card: GeForce RTX 2080 Ti). The molecular operating environment (MOE) 2022.02 ^31^ and Maestro (Schrödinger Release 2020–2022)^33^ were used as molecular modeling software. The crystal structures of the S protein in the open and closed conformations, along with LA in the S protein, were downloaded from the Protein Data Bank (PDB) (http://www.rcsb.org/ (accessed on 15 May 2023); PDB codes 7DF3, 6VYB, and 6ZB5).^3,21^ Missing loops in structures were constructed through homology modeling. A cubic water box with a 10 Å buffer distance was used for the solvation system between each box side and the protein atoms, with four sodium atoms used to neutralize the system. Before the MD simulation, the system was pre-equilibrated using a default relaxation routine implemented in Desmond. Three 100 ns MD simulations were performed, during which the equation of motion was integrated using a 2 fs time step in the NPT ensemble with the temperature (300 K) and pressure (1 atm) constant. All other parameters were set using the default Desmond default values. Data were collected every 1.2 ps (energy) and every 200 ps (trajectory). Each individual system was simulated in triplicate with a separate random seed as the starting point. Visualization of the S protein and MD trajectory analyses were carried out using Maestro. RMSD, RMSF, secondary structure, and protein–protein interaction analyses were performed using the Simulation Event Analysis tool and the Simulation Interaction Diagram of Desmond.

### Site Finder

The Site Finder application in MOE was used to find potential ligand binding pockets in generated S protein structures from frames in the molecular dynamics generated trajectory files. A 15-frame interval was applied, corresponding to 3 ns, and submitted to the Site Finder application, in PDB format. Site Finder produced a list of potential binding sites, including interacting residues and the size of the predicted pocket. Each pocket identified was visually analyzed, and pockets similar to the FABP were selected. The procedure was repeated in both open and closed conformations and, for each of the three FABP, in each S protein structure.

### Virtual Screening

The Enamine library of commercially available drug candidates was screened against the fatty acid binding pocket using the PDB ID 6ZB5 crystal structure (http://www.rcsb.org/ (accessed on 15 May 2023); 6ZB5).^21^ The structures of the compounds analyzed were built in MOE2019.10, saved in .sdf format, and prepared using the Maestro LigPrep tool by energy minimizing the structures (OPLS_2005 force field) and generating possible ionization states at pH 7 ± 2, tautomers, all possible stereoisomers per ligand, and low-energy ring conformers. The protein was preprocessed with the MOE Protein Preparation tool, and the resulting protein–ligand complex was saved in .mae format and prepared using the Schrödinger Protein Preparation Wizard by assigning bond orders, adding hydrogens, and performing a restrained energy minimization of the added hydrogens using the OPLS_2005 force field. Additionally, the protein was also saved in .oedu format and .mol2 format to be used with scoring software ScorePose (OpenEye) and PLANTS, respectively.^35,36^ The Glide high-throughput virtual screening tool (HTVS) was used to virtually screen the commercial database against the binding site, followed by a docking procedure with Glide SP.^33^ A 15 Å docking grid was prepared using the cocrystallized LA as the centroid, in parallel for the three FABP in the S protein. The library was docked on the active sites using the Glide HTVS docking algorithm,^16^ keeping the default parameters, setting to three the number of output poses per input ligand to include in the solution. The top 50,000 compounds scored by Glide HTVS were selected for a new docking procedure with Glide SP (standard precision) docking algorithm, setting to three the number of output poses per input ligand to include in the solution and performing a postdocking minimization of each of the poses kept.^33^ The output poses were saved as mol2 files. Docking poses obtained were then rescored (maintaining the identified pose) using Glide XP, CHEMPLP (PLANTS), and OpenEye (ScorePose) scoring functions.^34–36^ Using a single docking program and scoring function might introduce potential bias, which justified the use of three programs for rescoring. The value of each scoring function for each docking pose was then combined (consensus score), and only docking poses falling in the top 25% of the score value range for all four scoring functions were selected for visual inspection in the three FABP. The docking results were visually inspected in MOE 2022.02. The docking poses of the compounds obtained from the visual inspection were evaluated considering the following criteria: the ability of a compound to adequately occupy the fatty acid binding site (similar to LA); interactions predicted between the compound and protein residues defining the site. Given that LA has been confirmed as a ligand and has shown antiviral activity in experimental assays, docked molecules were superimposed with a crystallographic structure of LA bound to the S protein (PDB accession code 6ZB5). In the next step, the set of molecules from each pocket was combined, with only molecules capable of good predicted interactions in all pockets selected for further stages. Finally, the set of molecules identified for experimental validation was reduced to 20, by applying the Lipinski rule of five (selecting for good medicinal chemistry properties) and the SWISS-ADME webtool, to screen compound potential for toxicity (PAINS and Brenk analysis).^38^

### Source of Small Molecules

All of the compounds in this study, both the initial screened set and analogue compounds, were purchased from Enamine, Ltd. (Kyiv, Ukraine). The library used for the virtual screening study was the Enamine Screening Collection.^32^ Additionally, the analogue compounds were selected based on a similarity search on the Enamine REAL database, through which it was purchased.^48^ Molecular formulas (SMILES), molecular weight, and PAINS and Brenk analysis of tested compounds are reported in the Supporting Information (Table S1).

### Biological Assays

#### Binding Assays

An inhibitor screening assay kit was used to screen inhibitors of the S-ACE2 interaction (BPS Bioscience catalog no. 78012).^49^ The kit includes the S protein in its native trimeric conformation from the Wuhan strain, providing the best physiologically relevant model for this interaction.^50^ The assay kit also contains biotinylated-ACE2, streptavidin-HRP, and the assay buffers. The assay procedure was performed as follows: SARS-CoV-2 S protein was first coated onto a 96-well plate. Following this, biotinylated-ACE2 was incubated with the S protein on the plate and streptavidin-HRP was added to the plate. The interaction between biotinylated-ACE2 and SARS-CoV-2 S protein was then detected using a colorimetric substrate. The resulting color change was quantified by measuring the absorbance using a UV/VIS microplate reader. Compounds were dissolved in DMSO and diluted until a testing concentration of 200 μM was reached, with each compound tested in triplicate. Finally, one negative control (vehicle, 2% DMSO) and two positive controls were used, LA and PEA.

#### Cell Culture

Vero E6 cells used for cytotoxicity and antiviral assays were cultivated in Dulbecco’s modified Eagle medium (DMEM) with 10% FCS and 1% penicillin G/streptomycin (P/S) and grown in standard culture conditions, namely, in a humidified incubator at 37° and 5% CO_2_. Cells were evaluated daily, and the culture medium was changed whenever necessary, with cell passaging performed when desirable confluences of 70–80% were observed.

#### Cytotoxicity: Presto Blue Viability Assay

The Presto Blue assay was performed to determine the cytocompatibility between the cellular system and the test compounds. This assay is based on a ready-to-use, commercially available water-soluble preparation and allows a live-cell evaluation. The resazurin solution was used to assess cell viability based on the mitochondrial metabolization of this substance solution. Viable cells reduce the phenoxazine dye (resazurin), which results in color modification from blue to reddish over time that can be not only directly observed but also quantitively measured by UV–VIS spectrophotometry, functioning as a cell viability indicator. Vero E6 cells were seeded over a 96-well plate and maintained in incubation overnight (standard culture medium, 37 °C, 5% CO_2_ environment, and 80% humidified atmosphere). To perform the Presto Blue assay, the culture medium was removed from each well at every time point (24, 28, and 96 h) and replaced by a complete medium with 10% (v/v) of 10 Presto Blue cell viability reagent (Invitrogen, A13262, Thermo Fisher Scientific, Waltham, MA USA). To perform the analysis, cells were incubated for 60 min under standard conditions to allow metabolization of the reagent. The supernatant medium was then collected and transferred to a 96-well plate, and absorbance was read at 570 and 595 nm in a Multiskan FC microplate photometer (51119000, Thermo Fisher Scientific, Waltham, MA USA). Afterward, wells were washed with Dulbecco’s phosphate-buffered saline solution (DPBS, Gibco, 14190169) until the Presto Blue sediments were removed. Then, a fresh culture medium was added to each well, according to the time point specifications. A regular growth medium was used until the first time point (24 h), when it was replaced with DMEM (10% FCS, 1% P/S) supplemented with the test compound (determination of acute cytotoxicity). At the second time point (28 h), the medium was replaced with DMEM (2% FCS, 1% P/S) supplemented with test compounds and then left for 72 h until the last time point was reached (96 h) (determination of acute cytotoxicity). For the Presto Blue assessment, both control group and test compounds were considered, and for each group, blank wells (without cell seeding) were included. The wavelength for excitation was 570 nm, and that for emission was 595 nm. For that reason, the value obtained at 595 nm was subtracted from the value obtained for 570 nm (normalized value) for each well. In addition, the corrected absorbance for each experimental well, only considering seeded wells, was obtained by the subtraction of blank wells average from the normalized values of the respective sample group. The absorbance values were measured in triplicates. Data were further processed and normalized to the mean of the gold standard group and presented in a ratio between the 24 h time point and both 28 and 92 h time points, representing variation against initial cell viability as a baseline. Statistical analysis was performed with a one-way ANOVA, with Dunnett’s post hoc test.

#### SARS-CoV-2 Antiviral Assay: Virus Infection

Vero E6 cells were seeded into 96-well plates at a density of 40,000 cells per well in 100 μL of DMEM supplemented with 10% FCS and 1% P/S. Blank and vehicle controls were included. The cells were incubated overnight at 37 °C with 5% CO_2_ to reach approximately 70–80% confluence. Serial dilutions of the test compounds were prepared in DMEM (2% FCS, 1% P/S), with nine 3-fold dilutions ranging from 200 to 0.03 μM. The cells and compound solutions were then transferred to a biosafety level 3 laboratory for infection with SARS-CoV-2. The growth medium in each well was replaced with test compound-containing solutions immediately before infection. The top half of each plate received an additional mock DMEM (2% FCS, 1% P/S), while the bottom half received a virus-containing solution. Hence, cell infection was performed in two sets: (1) Vero E6 cells treated with test compounds and infected with SARS-CoV-2 and (2) Vero E6 cells treated with the test compound only, allowing a direct comparison of cell viability in the presence or absence of virus. Cell viability was compared with mock-treated samples treated with the vehicle only (0.5% DMSO [vol/vol]). Two controls were used (with an additional blank without cells to measure background signal): a positive control where cells were infected without the test compound and a negative control with noninfected cells treated with DMSO only (vehicle). The cells were infected with 0.05 MOI (multiplicity of infection) of SARS-CoV-2 strain INMI1 P4. The virus was obtained from EVAg.^51^ Additionally, the antiviral assays were also performed with other variants of concern, particularly the SARS-CoV-2 Delta and SARS-CoV-2 Omicron, obtained through private collaboration. The mock-treated and infected cells were incubated at 37 °C with 5% CO_2_ for 72–96 h, until a cytopathic effect (CPE) was observed in all control wells. CPE and potential aggregation effects by tested compounds were evaluated by optical microscopy. Upon CPE detection, some of the supernatant was harvested and frozen for downstream analyses. Cells and the remaining SN were frozen for viability assays.

#### SARS-CoV-2 Antiviral Assay: Cytotoxicity and Cell Viability

Cell viability was determined using CellTiter-Glo (Promega), which quantifies the amount of ATP present in the sample provided. ATP is a measure of metabolically active cells, allowing for the determination of the number of viable cells. Cells/SN that were treated with different concentrations of compounds and infected were thawed after being frozen at −80 °C and equilibrated to room temperature. In a white bottom plate (PerkinElmer 1/2 Area ViewPlate), 10 μL of lysed cells/medium was mixed with 10 μL of the CellTiter-Glo reagent (Promega). The plate was then mixed for 2 min in an orbital shaker to induce cell lysis and allowed to incubate at room temperature for 10 min to stabilize the luminescence signal. The luminescence signal was then measured with a plate reader (VICTOR Nivo, PerkinElmer). Antiviral activity is measured by the ability to reduce the viral effects, which reduce the number of viable cells and cause CPE. As a result, the ATP levels detected are also reduced along with the luminescence signal. This was expressed as the percentage of inhibitory effects of viral induced reduction of signal compared against untreated virus-infected positive control cells (100% CPE).

$$\mathrm{inhibitory}\;\mathrm{activity}=\frac{\left[(\mathrm{cells}+\mathrm{virus}+\mathrm{antiviral})-(\mathrm{cells}+\mathrm{virus})\right]}{\left[(\mathrm{cells})-(\mathrm{cells}+\mathrm{virus})\right]}$$

When 50% inhibition of viral induced reduction of CPE is detected, the antiviral activity is expressed by half-maximal effective concentration (EC_50_), the concentration of compound that achieves 50% of viral effects.

#### TCID_50_ Determination: Virus Titration

To determine the 50% tissue culture infectious dose (TCID_50_), a standard virus titration assay was performed. As previously, Vero E6 cells were seeded in a 96-well plate at a density of 40,000 cells per well and incubated overnight at 37 °C with 5% CO_2_. Six serial 10-fold dilutions of the virus supernatant were prepared in DMEM (2% FCS, 1% P/S). The supernatant used corresponds to the compound concentration with the highest activity and reduced cytotoxicity. Each dilution was added to six wells, starting with the lowest dilution. The plates were incubated at 37 °C with 5% CO_2_ for 96 h, during which CPE was monitored daily. Following the incubation period, each well was scored for the presence or absence of CPE. TCID_50_ can then be calculated using the Reed–Muench method, which involves determining the dilution at which 50% of the wells show CPE. This is done by plotting the number of positive wells against the dilution factor and finding the dilution with 50% probability of infection. Alternatively, cell viability can be determined with CellTiter-Glo and the virus titer causing 50% reduction in CPE can be determined.

#### RT-ddPCR

RT-ddPCR was employed to quantify SARS-CoV-2 with high precision and sensitivity. The RT-ddPCR reactions were prepared using a One-Step RT-ddPCR Advanced Kit for Probes (Bio-Rad), with target-specific primers and probes (sequence, forward primer: ACAGGTACGTTAATAGTTAATAGCGT; reverse primer: ATATTGCAGCAGTACGCACACA; probe sequence: ACACTAGCCATCCTTACTGCGCTTCG).^52^ Each 20 μL reaction mixture contained 5 μL of a Supermix, 2 μL of reverse transcriptase (RT), 1 μL of a primer/probe mix, and 11 μL of an inactivated viral sample from the dilution with the highest activity for each compound. Droplets were generated by using a Bio-Rad QX200 droplet generator. Briefly, the reaction mixture was loaded into the sample wells of a DG8 cartridge, along with ddPCR droplet reader oil for probes in the oil wells and covered by DG8 gaskets. The cartridge was then placed into the QX200 droplet generator. After droplet generation, the emulsified PCR reactions were transferred to a 96-well PCR plate and sealed with a foil seal using a PX1 PCR plate sealer. PCR amplification was carried out using a thermal cycler with the following conditions: initial cDNA synthesis from an RNA template by RT at 50 °C for 15 min, followed by DNA polymerase activation at 95 °C for 2 min, and finally by 40 cycles of 95 °C for 15 s and 60 °C for 30 s. The droplets were then read using a QX200 droplet digital system, which counted the number of fluorescent-positive and fluorescent-negative droplets to determine the absolute quantity of target molecules using Poisson distribution analysis. Data analysis was performed using the manufacturer’s software, for absolute quantification of the SARS-CoV-2 genome copies per 20 μL of the initial diluted sample.

## Supplementary Material



## Notes

Chemical structures of all compounds reported, molecular weight, calculated LogP, H-bond-accepting groups, H-bond-donating groups, and PAINS and Brenk analysis; cytotoxicity results and SARS-CoV-2 Omicron infection cell viability (PDF)

## Abbreviations

ACE2: angiotensin converting enzyme 2
ADME: absorption, distribution, metabolism, excretion
CC_50_: concentration 50% cytotoxic
COVID-19: coronavirus disease 19
CPE: cytopathic effects
DMEM: Dulbecco’s modified Eagle medium
DMSO: dimethylsulfoxide
FABP: fatty acid binding pocket
FCS: fetal calf serum
LA: linoleic acid
MD: molecular dynamics
MERS-CoV: Middle Eastern respiratory syndrome coronavirus
P/S: penicillin G/streptomycin
PEA: palmitoylethanolamide
RBD: receptor binding domain
RMSD: root-mean-square deviation
RMSF: root-mean-square fluctuation
RT- ddPCR: digital droplet PCR
S protein: glycosylated spike protein
SARS-CoV-2: severe acute respiratory syndrome coronavirus 2
SARS-CoV: severe acute respiratory syndrome coronavirus
TCID_50_: 50% tissue culture infectious dose
TMPRSS2: transmembrane protease serine 2
