# Supplementary material for: In Silico Discovery of a Novel Antiviral Scaffold for SARS-CoV‑2 Targeting the Spike Glycoprotein through the Fatty Acid Binding Pocket
Source: ACS Omega. 2025 Jun 4;10(23):24117–32. doi: 10.1021/acsomega.4c10519 (PMC12177760; doi:10.1021/acsomega.4c10519)
Supplement: Supplementary file 1 [file ao4c10519_si_001.pdf]

In silico discovery of a novel antiviral scaffold for SARS-CoV-2 targeting the spike glycoprotein through the fatty acid binding pocket

Luís Queirós-Reis[1], Mari Kaarbø[2], Huda Al-Baldawi[3], Rui Alvites[1,4,5,6], Ana Colette Maurício[1,4,5], Andrea Brancale[7], Marcella Bassetto[8,9], João R. Mesquita [1,10]*

1. Abel Salazar Institute of Biomedical Sciences (ICBAS), University of Porto, 4050-313 Porto, Portugal; up201205115@up.pt (L.Q.-R.)

2. Department of Microbiology, Oslo University Hospital, Oslo, Norway

3. Department of Microbiology, University of Oslo, Oslo, Norway

4. Animal Science Study Centre (CECA), University of Porto Agroenvironment, Technologies and Sciences Institute (ICETA), 4051-401 Porto, Portugal

5. Associate Laboratory for Animal and Veterinary Science (AL4AnimalS), 1300-477 Lisboa, Portugal

6. University Institute of Health Sciences (CESPU), Avenida Central de Gandra 1317, 4585-116 Gandra, Portugal

7. University of Chemistry and Technology, Prague 166 28 Praha, Czechia; andrea.brancale@vscht.cz

8. School of Pharmacy and Pharmaceutical Sciences, College of Biomedical and Life Sciences, Cardiff University, Cardiff, CF10 3BN, UK; bassettom1@cardiff.ac.uk

9. Department of Chemistry, Faculty of Science and Engineering, Swansea University, Swansea, SA2 8PP, UK

10. Epidemiology Research Unit (EPIunit), Institute of Public Health, University of Porto, 4050-091 Porto, Portugal

*Correspondence: L.Q.-R., up201205115@up.pt

Table S1 - Chemical structures of compounds reported, including molecular Weight, calculated LogP, H-bond accepting groups, H-bond donating groups, as well as PAINS and BRENK analysis.

Molecule	SMILES	MW	PAINS	BRENK	H-bond acceptors	H-bond donors	LogP
Linoleic acid	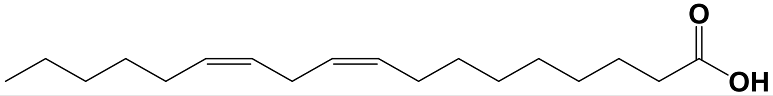	280.45	0	0	2	1	5.88
PEA	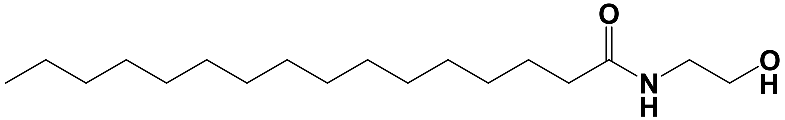	299.49	0	0	2	2	4.76
1	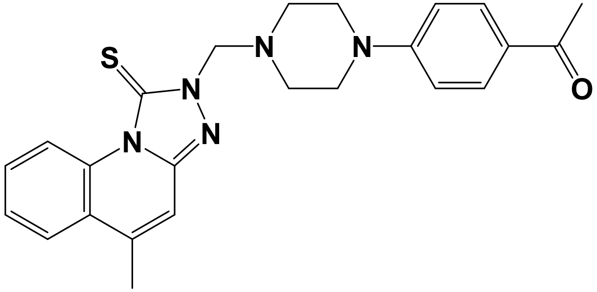	431.55	0	1	3	0	3.55
2	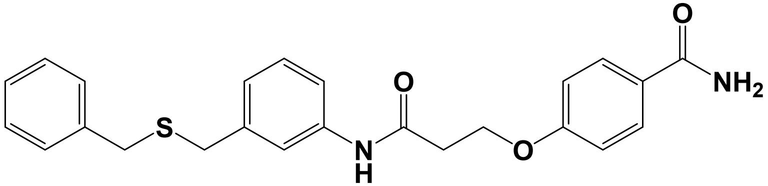	420.52	0	0	3	2	3.74

3	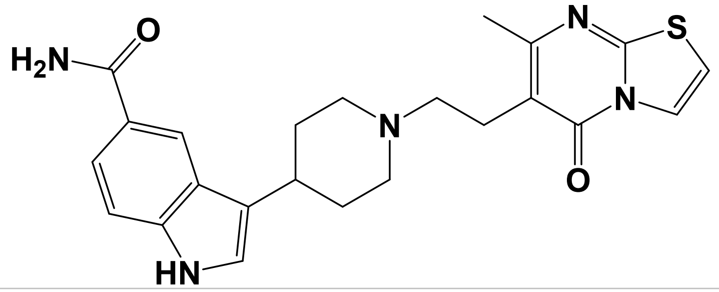	435.54	0	0	4	2	2.86
4	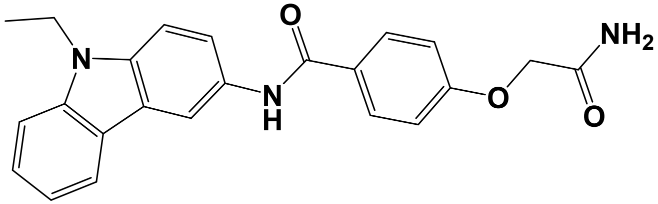	387.43	0	0	3	2	3.03
5	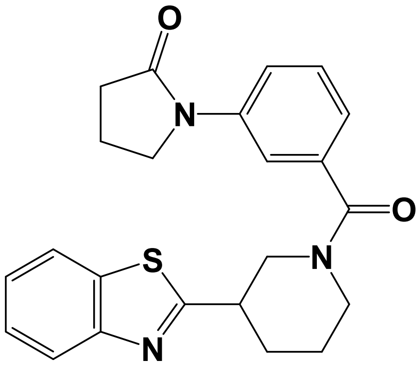	405.51	0	0	3	0	3.72
6	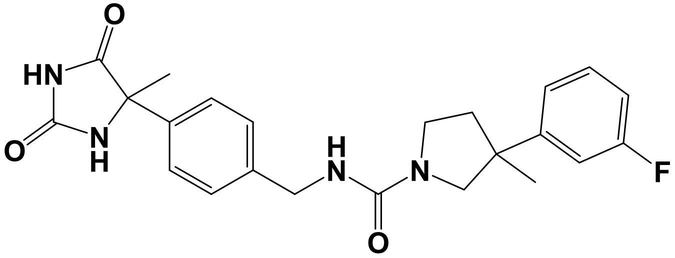	424.47	0	1	4	3	2.65

7	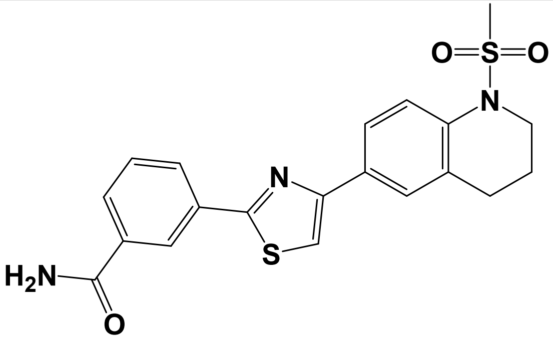	413.51	0	0	4	1	2.91
8	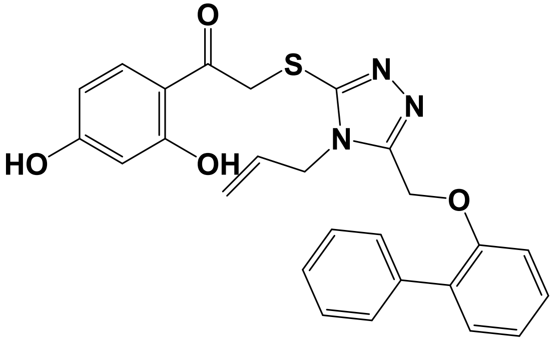	473.54	0	1	6	2	4.16
9	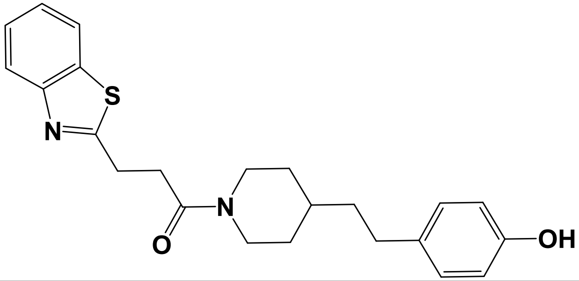	394.53	0	0	3	1	4.43

10	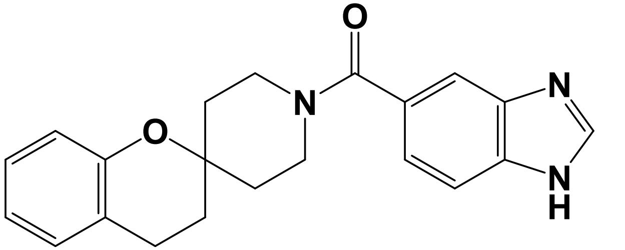	347.41	0	0	3	1	3.11
11	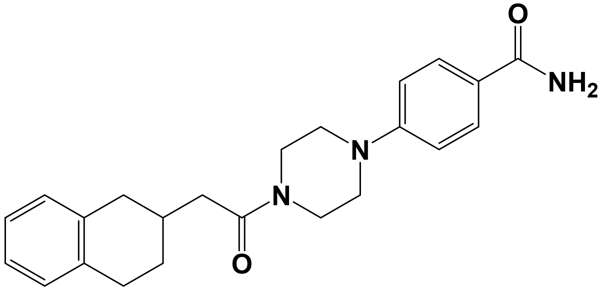	377.48	0	0	2	1	2.67
12	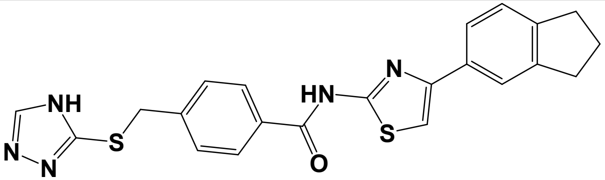	433.55	0	0	4	2	4.12
13	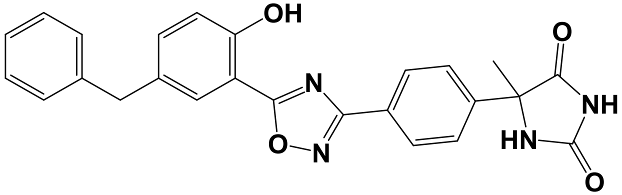	440.45	0	1	6	3	3.39
14	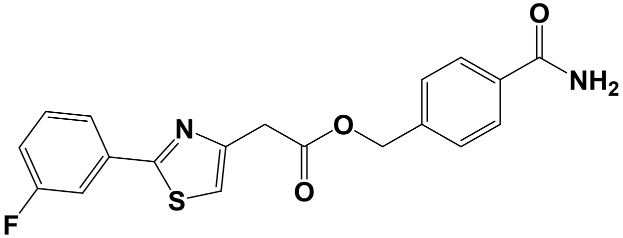	370.4	0	0	5	1	3.38

15	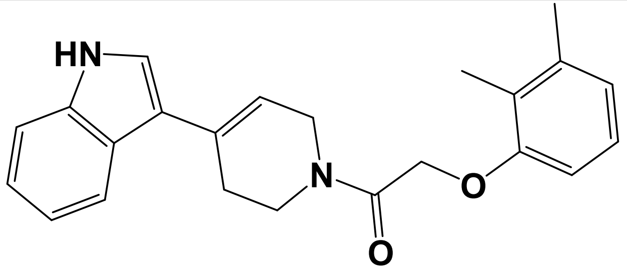	360.45	0	0	2	1	3.89
16	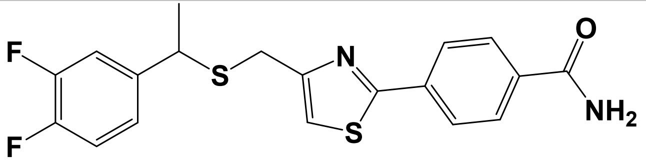	390.47	0	0	4	1	4.57
17	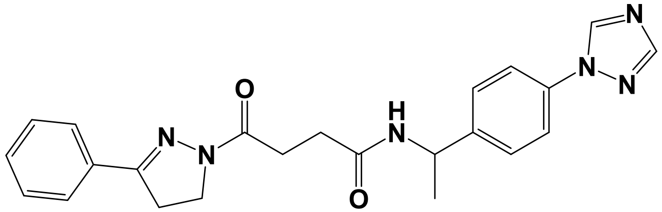	416.48	0	0	5	1	2.36
18	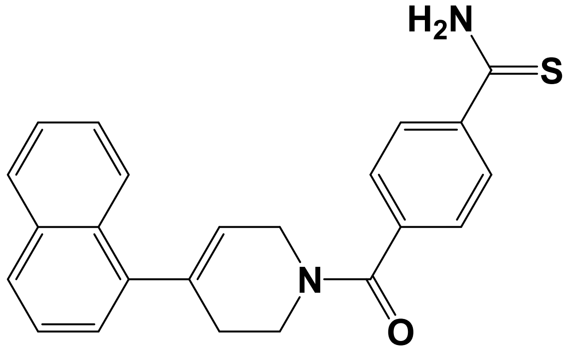	372.48	0	1	1	1	4.02
19	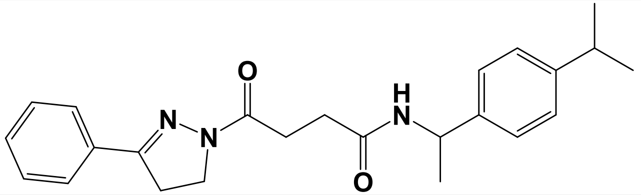	391.51	0	0	3	1	3.79

20	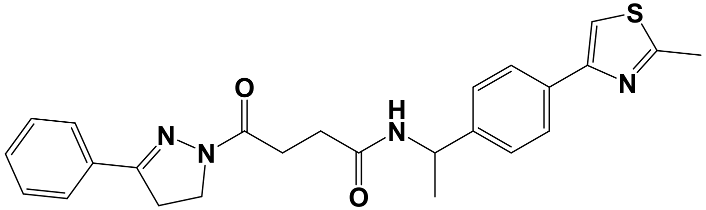	446.56	0	0	4	1	3.91
21	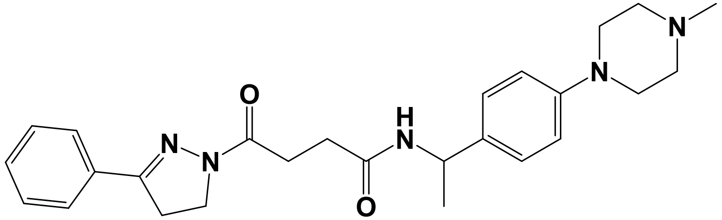	447.57	2	0	4	1	2.6
22	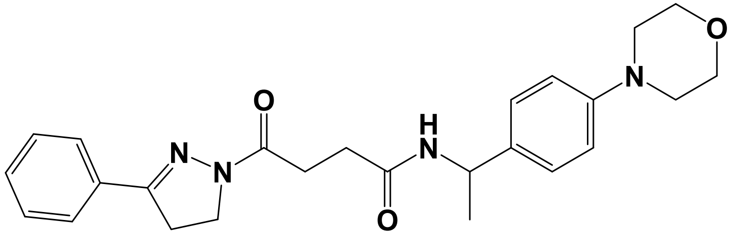	434.53	2	0	4	1	2.66
23	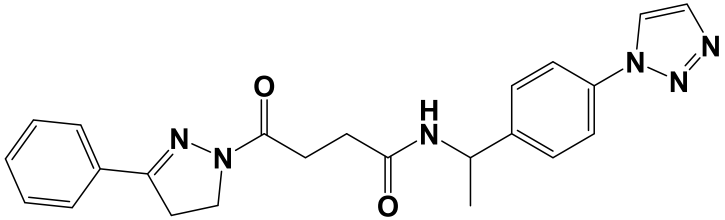	416.48	0	0	5	1	2.29
24	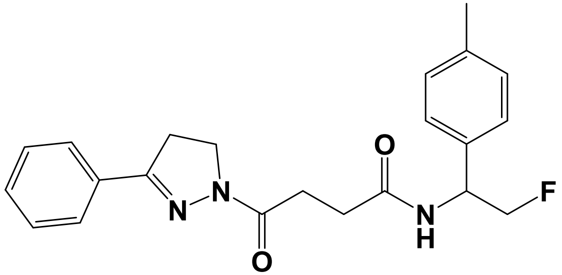	381.44	0	0	4	1	3.24

25	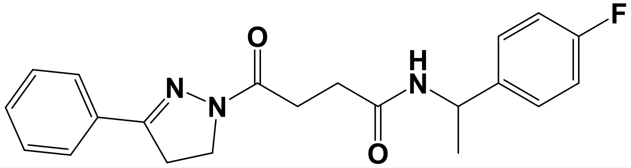	367.42	0	0	4	1	3.17
26	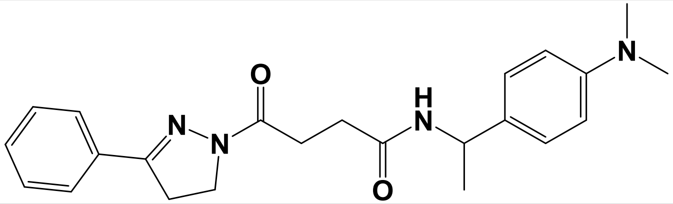	392.49	2	0	3	1	2.88
27	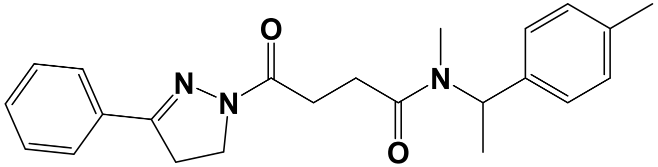	377.48	0	0	3	0	3.34
28	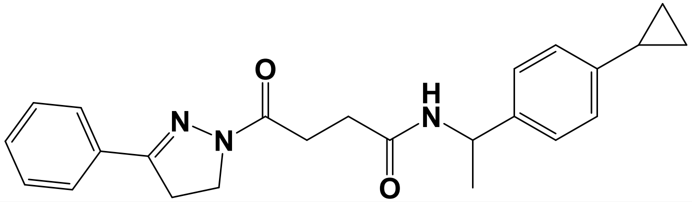	389.49	0	0	3	1	3.63
29	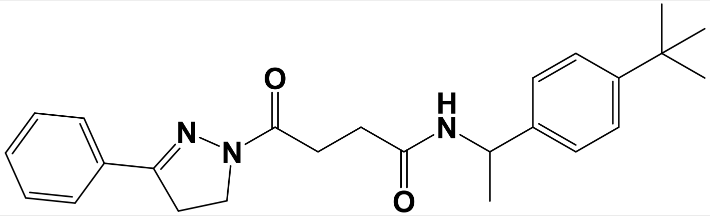	405.53	0	0	3	1	4.06

30	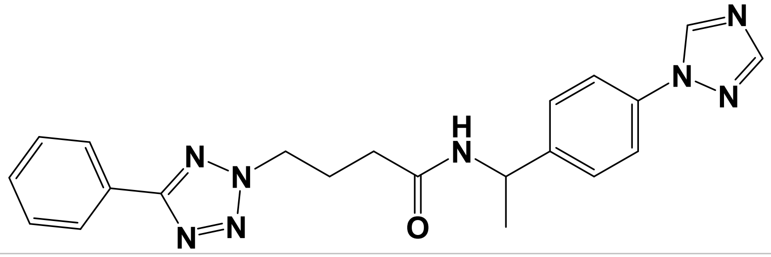	402.45	0	0	6	1	2.44
31	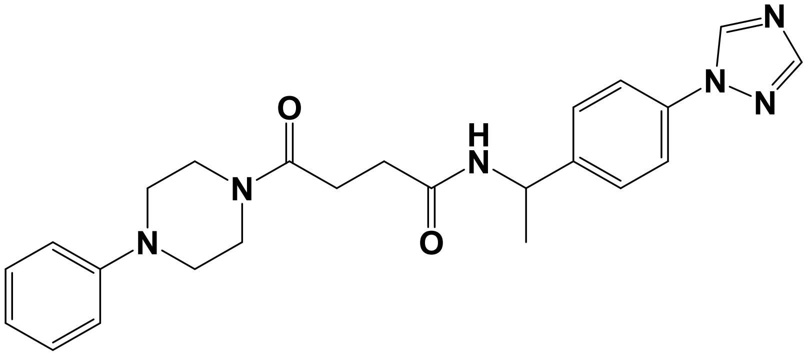	432.52	0	0	4	1	2.19
32	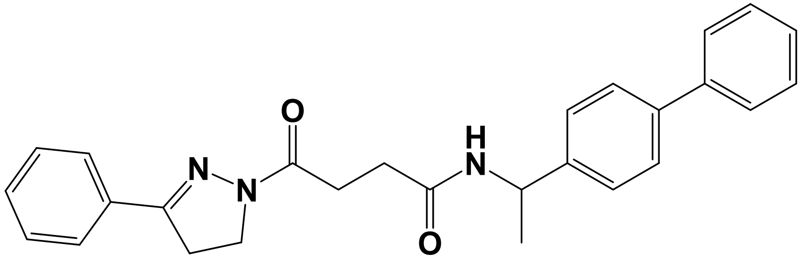	425.52	0	0	3	1	4.15
33	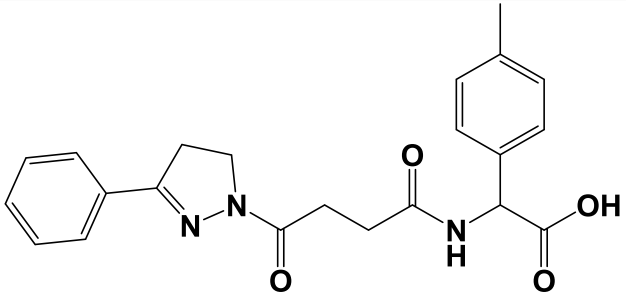	393.44	0	0	5	2	2.44

34	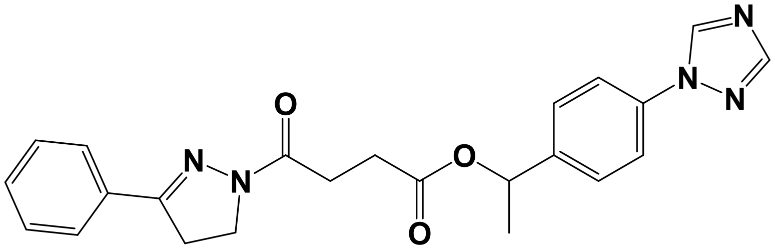	417.46	0	0	6	0	2.83
35	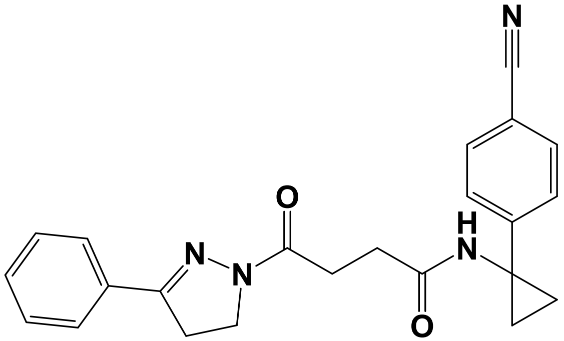	386.45	0	0	4	1	2.73
36	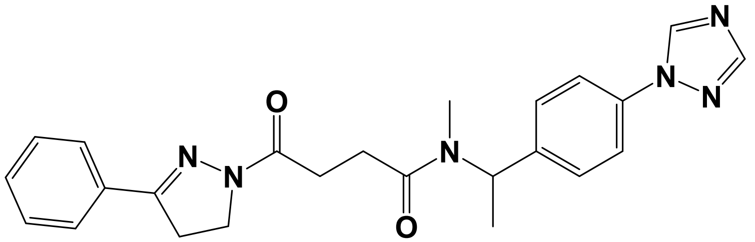	430.5	0	0	5	0	2.62
37	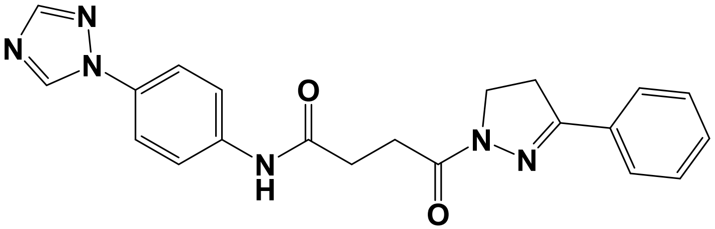	388.42	0	0	5	1	1.99

38	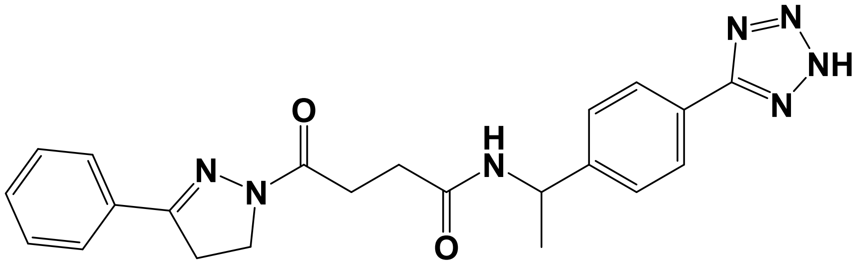	417.46	0	0	6	2	2.14

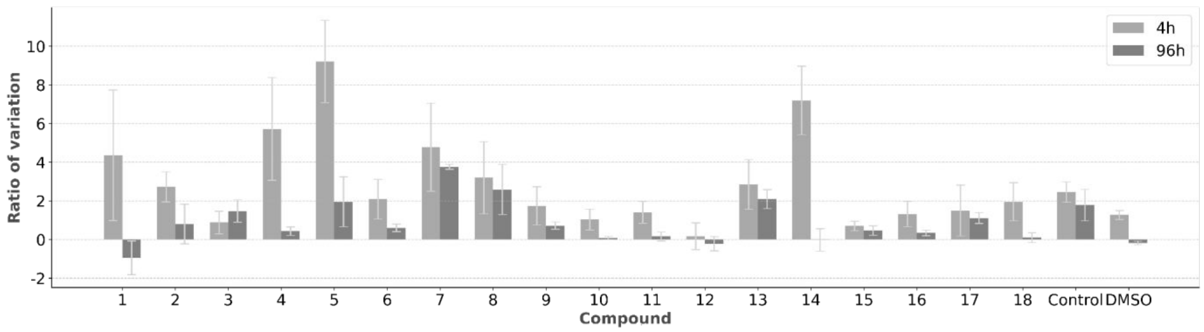

Figure S1 – Preliminary cytotoxicity evaluation performed on Vero E6 cells, with the screened compounds, a growth control and a cytotoxicity control. The initial growth at 0h is a baseline against which growth at 4h and 96h is compared, as observed in the experimental protocol for viral infection in the proceeding cells assays.

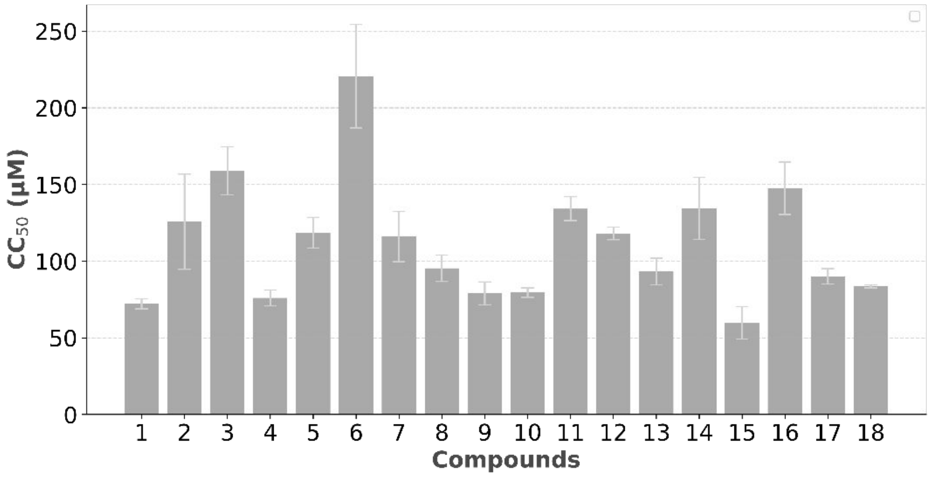

Figure S2. Cytotoxicity of screened compounds (X-axis) in Vero E6 cells, measured by the CellTiter-Glo® method. Cytotoxicity is represented by CC_{50} in the Y-axis, the concentration of test compounds required to reduce cell viability by 50%. The bars represent the mean ± SEM from three experimental repeats. Abbreviations: CC_{50} – cytotoxic concentration 50%.

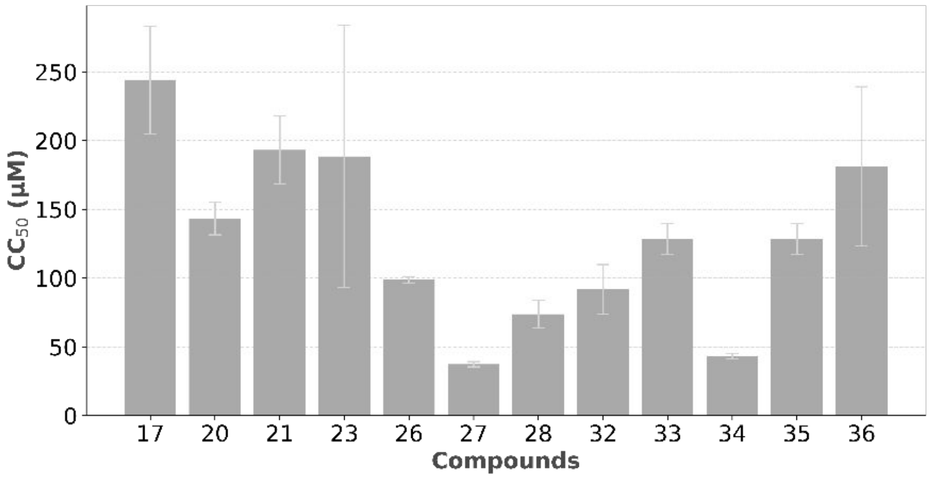

Figure S3. Cytotoxicity of screened compounds (X-axis) in Vero E6 cells, measured by the CellTiter-Glo® method. Cytotoxicity is represented by CC_{50} in the Y-axis, the concentration of test compounds required to reduce cell viability by 50%. The bars represent the mean ± SEM from three experimental repeats. Abbreviations: CC_{50} – cytotoxic concentration 50%.

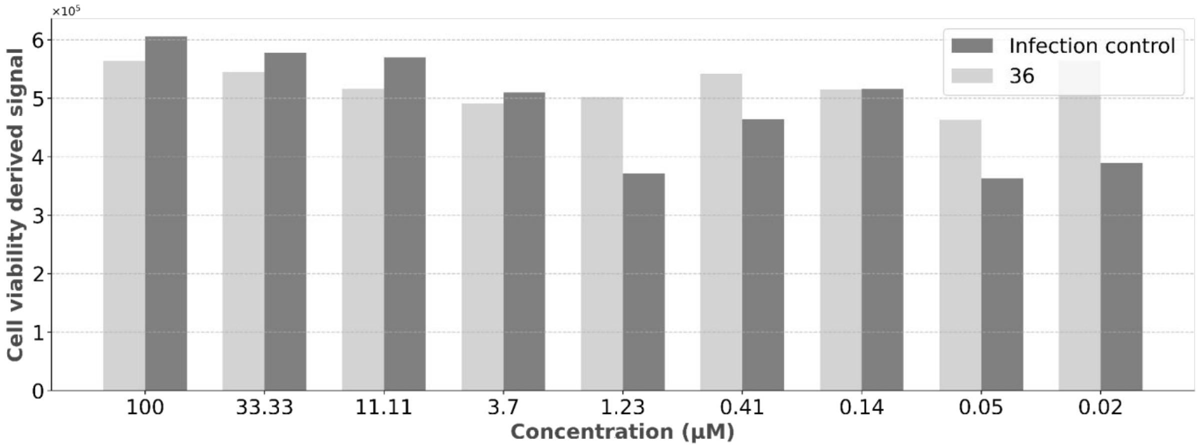

Figure S4 – Infection of Vero E6 cells with SARS-CoV-2 Omicron Variant at multiple concentrations. Cell viability was evaluated using the CellTiter-Glo® method.
